# A new bioavailable fenretinide formulation with antiproliferative, antimetabolic, and cytotoxic effects on solid tumors

**DOI:** 10.1038/s41419-019-1775-y

**Published:** 2019-07-23

**Authors:** Isabella Orienti, Federica Francescangeli, Maria Laura De Angelis, Katia Fecchi, Lucilla Bongiorno-Borbone, Michele Signore, Angelo Peschiaroli, Alessandra Boe, Alessandro Bruselles, Angelita Costantino, Adriana Eramo, Valentina Salvati, Giovanni Sette, Paola Contavalli, Lello Zolla, Toshihiko Oki, Toshio Kitamura, Massimo Spada, Alessandro Giuliani, Marta Baiocchi, Filippo La Torre, Gerry Melino, Marco Tartaglia, Ruggero De Maria, Ann Zeuner

**Affiliations:** 1 Department of Pharmacy and Biotechnology, University of Bologna via San Donato 19/2, 40127 Bologna, Italy; 2 Department of Oncology and Molecular Medicine, Istituto Superiore di Sanità, Viale Regina Elena 299, 00161 Rome, Italy; 3 Center for Gender-Specific Medicine, Istituto Superiore di Sanità, Viale Regina Elena 299, 00161 Rome, Italy; 4 Department of Experimental Medicine and Surgery, University of Rome Tor Vergata, Via Montpellier 1, 00133 Rome, Italy; 5 RPPA Unit, Proteomics, Core Facilities, Istituto Superiore di Sanità, Viale Regina Elena 299, 00161 Rome, Italy; 6 Core Facilities, Istituto Superiore di Sanità, Rome, Italy; 7 Department of Biomedical and Biotechnological Sciences BIOMETEC, University of Catania, via Santa Sofia 97, 95123 Catania, Italy; 8 DAFNE Department, University Tuscia, Via S. Camillo de Lellis, 01100 Viterbo, Italy; 9 Division of Cellular Therapy, The Institute of Medical Science, The University of Tokyo, Minato-ku, Tokyo 108-8639 Japan; 10 Division of Stem Cell Signaling, The Institute of Medical Science, The University of Tokyo, 4-6-1 Shirokanedai, Minato-ku, Tokyo 108-8639 Japan; 11 Center of Animal research and Welfare, Istituto Superiore di Sanità, Rome, Italy; 12 Environment and Health Department, Istituto Superiore di Sanita’, Rome, Italy; 13 Surgical Sciences and Emergency Department, Division of Emergency & Trauma Surgery, Emergency Department, Policlinico Umberto I/Sapienza University, Viale del Policlinico 155, 00161 Rome, Italy; 14 Genetics and Rare Diseases Research Division, Ospedale Pediatrico Bambino Gesù, Viale di San Paolo 15, 00146 Rome, Italy; 15 Institute of General Pathology, Catholic University of the Sacred Heart, Largo Francesco Vito 1, 00168 Rome, Italy

## Abstract

Fenretinide is a synthetic retinoid characterized by anticancer activity in preclinical models and favorable toxicological profile, but also by a low bioavailability that hindered its clinical efficacy in former clinical trials. We developed a new formulation of fenretinide complexed with 2-hydroxypropyl-beta-cyclodextrin (nanofenretinide) characterized by an increased bioavailability and therapeutic efficacy. Nanofenretinide was active in cell lines derived from multiple solid tumors, in primary spheroid cultures and in xenografts of lung and colorectal cancer, where it inhibited tumor growth independently from the mutational status of tumor cells. A global profiling of pathways activated by nanofenretinide was performed by reverse-phase proteomic arrays and lipid analysis, revealing widespread repression of the mTOR pathway, activation of apoptotic, autophagic and DNA damage signals and massive production of dihydroceramide, a bioactive lipid with pleiotropic effects on several biological processes. In cells that survived nanofenretinide treatment there was a decrease of factors involved in cell cycle progression and an increase in the levels of p16 and phosphorylated p38 MAPK with consequent block in G0 and early G1. The capacity of nanofenretinide to induce cancer cell death and quiescence, together with its elevated bioavailability and broad antitumor activity indicate its potential use in cancer treatment and chemoprevention.

Over the last decades, the use of targeted agents and immunotherapeutics has increasingly flanked classic cytotoxics, leading to a significant survival improvement for selected subsets of cancer patients. Nevertheless, new therapies are often associated to high economic burden, toxic side effects and absence of predictive biomarkers of efficacy. Therapy failure in cancer patients is tightly linked to the presence of tumor-initiating cells responsible for drug resistance and cancer relapse^1,2^. Therefore, effective therapeutic strategies should ideally be able not only to kill cancer cells but also to prevent the reactivation of remaining tumorigenic cells. Fenretinide (N-4-hydroxyphenyl-retinamide) is a synthetic derivative of all-trans retinoic acid characterized by high cytotoxic efficacy against cancer cells in vitro and previously investigated as a potential anticancer and chemopreventive drug^3–11^. Previous clinical phase I-III evaluations of fenretinide have shown minimal systemic toxicity and good tolerability^7,12,13^, fostering additional studies aimed at exploiting the selective anticancer effect of this compound. However, clinical trials aimed at evaluating the activity of fenretinide in cancer patients yielded frustrating results as therapeutic plasma levels could not be attained due to the poor aqueous solubility and consequent low bioavailability of the drug^3,8,13^. In fact plasma concentrations of fenretinide remained below the minimum threshold for the onset of the antitumor activity also after multiple and protracted administrations^3,13^. Formulations aimed at increasing fenretinide bioavailability were previously prepared by drug incorporation into lipid matrices or oil-in-water emulsions. They showed an improved performance compared to previous studies but faced some problems related to the increase of the administration dose and the correspondent increase in plasma concentration^14–17^. We have prepared a new fenretinide formulation characterized by improved aqueous solubility through drug salification and complexation with 2-hydroxypropyl β-cyclodextrin, a solubilizing excipient endowed with favorable biodistribution and reduced toxicity^18^. The new fenretinide formulation (referred thereafter as nanofenretinide, NanoFEN) showed high anticancer efficacy in vitro and in vivo against cell lines from multiple tumors, primary spheroid cultures of lung and colon cancer cells and tumor xenografts in the absence of macroscopic toxic effects. A global analysis of NanoFEN-activated events revealed a widespread inhibition of the mTOR pathway, cell death with mixed autophagic/apoptotic features and cell cycle block with induction of quiescence. Such events occurred in parallel with a massive accumulation of bioactive dihydroceramide lipids, pleiotropic inhibitors of cell cycle and metabolism. Altogether, these results indicate that NanoFEN activates a multifactorial program in cancer cells composed by signals of apoptosis, autophagy and proliferative/metabolic inhibition, resulting in a widespread and durable antitumor effect. Although additional studies will be required to establish the efficacy and absence of toxicity of NanoFEN in human subjects, our results indicate this compound as a candidate for future clinical studies.

## Results

### Generation of a new bioavailable fenretinide formulation

**Fig. 1:**
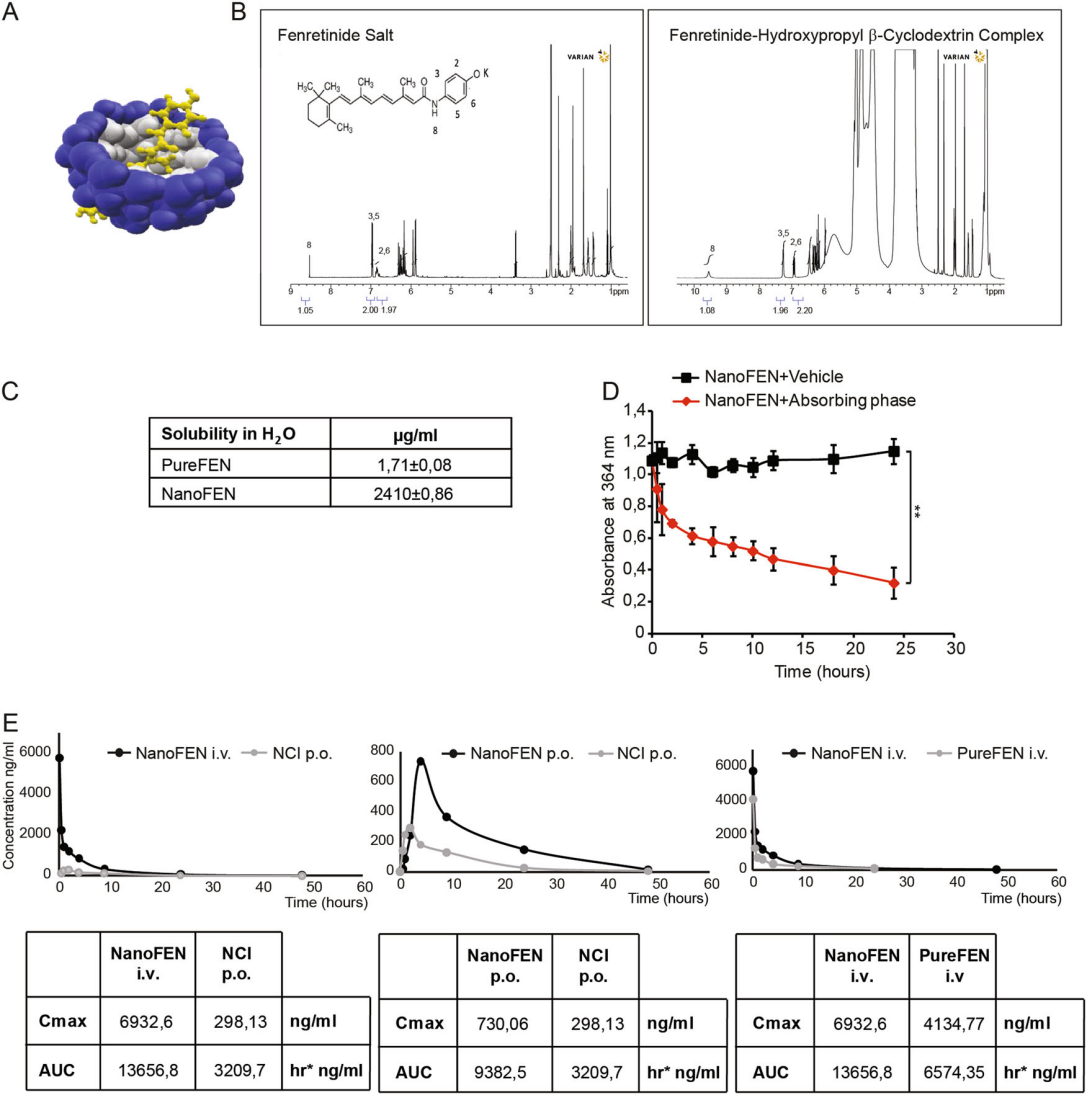
Generation and analysis of a new bioavailable fenretinide formulation. **a** Tridimensional representation of fenretinide (yellow) complexed with 2-hydroxypropyl-β-cyclodextrin (blue). **b** ^1^H NMR analysis of fenretinide salt (left panel) and fenretinide complex with cyclodextrin (right panel). Chemical shift changes corresponding to protons H8, H3, H5, H2, H6 of the complexed fenretinide (right panel). **c** Solubility of PureFEN and NanoFEN in water at 25 °C. **d** Release of fenretinide from NanoFEN in a plain aqueous environment (black squares, NanoFEN+Vehicle) or in the presence of an absorbing phase (red diamonds, NanoFEN+Absorbing phase). Values represent mean ± SD of three independent experiments. **P < 0.01 from two-tailed *t*-test. **e** Plasma pharmacokinetic profiles of fenretinide after single administration of 5 mg/kg from different formulations: left panel, NanoFEN i.v. (black circles) compared to NCI p.o. (grey circles); central panel, NanoFEN p.o. (black circles) compared to NCI p.o. (grey circles); right panel, NanoFEN i.v. (black circles) compared to PureFEN i.v. (grey circles). Tables show the Cmax and AUC values of the relative graphs

The extremely poor aqueous solubility of fenretinide traditionally represents a strong drawback limiting the bioavailability of this molecule and its use in anticancer therapy. A possible solution for increasing fenretinide bioavailability is its complexation with cyclodextrins, in order to improve the drug aqueous solubility and provide chemical stability. Compared to other solubilizing excipients marketed in pharmaceutical products, cyclodextrins are endowed with favorable biodistribution and reduced toxicity also after repeated administrations^18^. However, the very low solubility of fenretinide in water prevents its spontaneous inclusion into the hydrophobic cavity of the cyclodextrin, compromising the effectiveness of the complexation process. We overcame this obstacle by rising the aqueous solubility of fenretinide through an unprecedented procedure of fenretinide salification that allowed drug complexation to take place in the presence of hydroxypropyl β-cyclodextrin (Fig. 1a). The formation of the fenretinide-cyclodextrin complex is demonstrated by the chemical shift changes in the nuclear magnetic resonance (^1^H NMR) spectrum corresponding to protons H8, H3, H5, H2, H6 of the complexed fenretinide (Fig. 1b, right panel) with respect to the fenretinide salt (Fig. 1b, left panel). Figure 1c reports the aqueous solubility of NanoFEN, showing it is >1000 times higher than pure fenretinide (PureFEN). Importantly, in order to provide an effective antitumor activity, the NanoFEN complex should be stable in aqueous body fluids and ideally release its active molecule only after accumulation in the tumor site, behaving as an hydrophobic absorbing phase and favouring accumulation by the enhanced permeability and retention effect (EPR effect)^19^. Figure 1d shows that the release of fenretinide from the complex takes place only in the presence of an adsorbing hydrophobic phase while it is suppressed in a plain aqueous environment, thus supporting a productive use of this formulation in the antitumor therapy. Pharmacokinetics and bioavailability of NanoFEN in mice were assessed by comparing intravenous administration of NanoFEN *versus* fenretinide contained in the oral gelatin capsules provided by the National Cancer Institute and previously used in the majority of clinical trials, which contain fenretinide dispersed in corn oil and polysorbate-80 (referred thereafter as NCI fenretinide). We compared intravenous administration of NanoFEN *versus* oral administration of NCI fenretinide (Fig. 1e, left), oral administration of NanoFEN *versus* oral administration of NCI fenretinide (Fig. 1e, center) and intravenous administration of NanoFEN *versus* intravenous administration of PureFEN (Fig. 1e, right). The latter was dissolved in 10% ethanol and saline to obtain drug solubilization, although this procedure could not be applicable to human use due to the presence of fenretinide precipitates. In all three cases, NanoFEN provided significantly higher drug exposure, being Area Under the Curve (AUC) values obtained after NanoFEN administration always higher than those obtained with other systems either by oral or intravenous route (Fig. 1e and Table 1). Previous formulations of fenretinide used in clinical trials were characterized by a favorable toxicological profile, with side effects generally limited to diminished dark adaptation and dermatologic disorders even at high dosages^7,12,13,20,21^. In order to determine whether NanoFEN may produce macroscopic side effects in mice, we evaluated body weight, hepatic and hematologic parameters in mice treated intravenously with NanoFEN. Body weight, alanine transaminase (ALT), aspartate transaminase (AST), bilirubin and hematologic values at the end of the treatment were comparable to controls, providing preliminary evidences on the absence of systemic toxicity, hepatic damage and myelosuppression (Supplementary Fig. 1a–c). These results show that the new physico-chemical properties of NanoFEN provide a substantial increase in fenretinide plasma levels without macroscopic side effects, although in-depth toxicological analyses will be required to rule out potential organ-specific toxicities.

**Table 1:** Main pharmacokinetic parameters of Fenretinide in CD1 female mice after administration of Nanofenretinide by oral or intravenous route in comparison with the NCI capsules and pure Fenretinide administered at the same dose (5 mg/Kg fenretinide)

Pk parameters	NanoFEN i.v.	PureFEN i.v.	NanoFEN p.o.	NCI p.o.	Units
AUC 0-last	13563.5	6420.32	9378.9	3201	h*ng/ml
AUC inf	13656.8	6574.35	9382.5	3209.7	h*ng/ml
Ke	0.094	0.088	0.106	0.089	1/h
HL	7.41	7.87	6.54	7.79	h
Tmax	–	–	4	2	h
Vd	3.9	6.52	–	–	L/kg
Cmax	6932.6	4134.77	730.06	298.13	ng/ml
Cl	0.366	0.574	–	–	L/hr/kg

### NanoFEN-induced killing of cancer cells from solid tumors

**Fig. 2:**
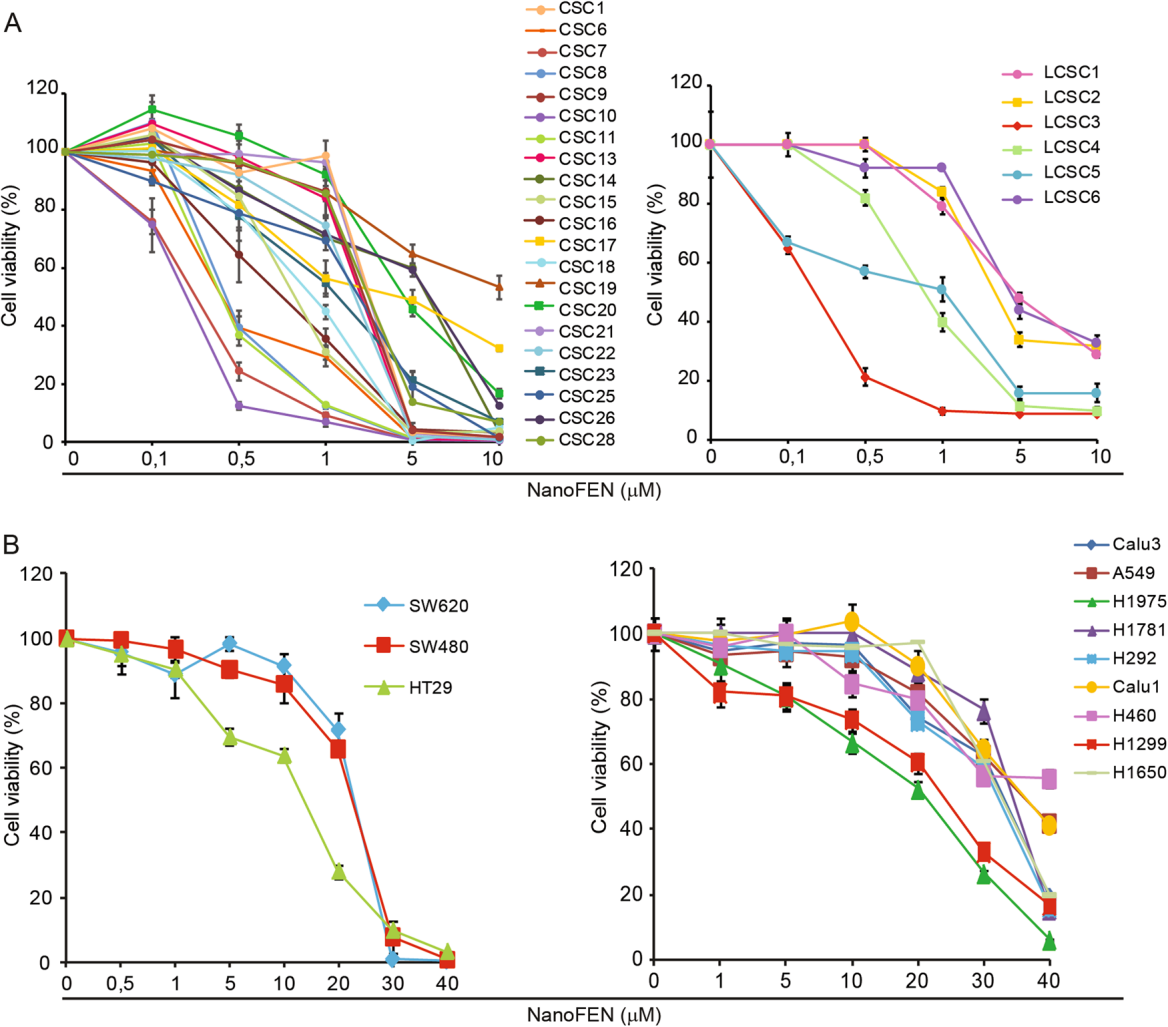
Effects of NanoFEN on lung and colorectal cancer cells. **a** Cell viability of CRC spheroid cells (CSCs) (left) and lung cancer spheroid cells (LCSCs) (right) treated with NanoFEN at the indicated concentrations. **b** Cell viability of CRC commercial cell lines (left) and lung cancer commercial cell lines (right) treated with NanoFEN at the indicated concentrations. Values represent mean ± SD of three independent experiments

Multicellular spheroid cultures derived from primary tumors have been shown to constitute a reliable system for drug testing as they provide a closer model of cancer heterogeneity as compared to commercial cell lines^22–25^. Primary spheroid cultures were derived from both lung and colorectal cancer (CRC) patients, biobanked at early passages after isolation, characterized for common genetic mutations and validated for their ability to reproduce the histology of the original tumor in mouse xenografts^25,26^. We tested NanoFEN on twenty-one spheroid lines of CRC and six spheroid lines of lung cancer, the latter being representative of all the major histological tumor types (adenocarcinoma, squamous cell carcinoma, large cell neuroendocrine carcinoma). NanoFEN was able to induce death of colon and lung spheroids, with the latter showing a sensitivity related to tumor histotype (Fig. 2a and Supplementary Fig. 2a, b), although an analysis on a larger number of samples would be required to confirm this correlation. Notably, the sensitivity of colorectal spheroids to NanoFEN was independent of the presence of common mutations incompatible with anti-EGFR agents, suggesting that patients not eligible for targeted therapies could in principle benefit from NanoFEN treatment. NanoFEN was also tested on three ATCC CRC cell lines and nine ATCC lung cancer cell lines, where it induced cell death with higher IC50 as compared to primary spheroid lines (Fig. 2b and Supplementary Fig. 2a, b). We reasoned that the presence of 10% serum in the culture media of ATCC lines may account for their lower sensitivity to NanoFEN as compared to spheroid lines (which are grown in serum-free media). Thus, we treated ATCC cells with NanoFEN in the presence of 1% serum and noticed that the IC50 was affected in CRC cell lines but not in lung cancer cell lines (Supplementary Fig. 2a, b). Thus, the differential sensitivity of ATCC lines and of spheroid lines likely depends on a combination of culture conditions and of yet unidentified factors. Finally, we tested the effect of NanoFEN on primary cultures of melanoma, breast cancer, glioblastoma and sarcoma, and on ATCC lines of hematologic cancer cells. NanoFEN displayed a broad spectrum cytotoxic effect on all the cells tested and particularly on primary cultures, which displayed IC50 values ranging from 0.2 to 4.9 µM (Supplementary Fig. 2c–g). These results suggest that NanoFEN interferes with mechanisms shared by multiple tumors to inhibit cell viability.

### In vivo antitumor efficacy of NanoFEN on lung and CRC xenografts

**Fig. 3:**
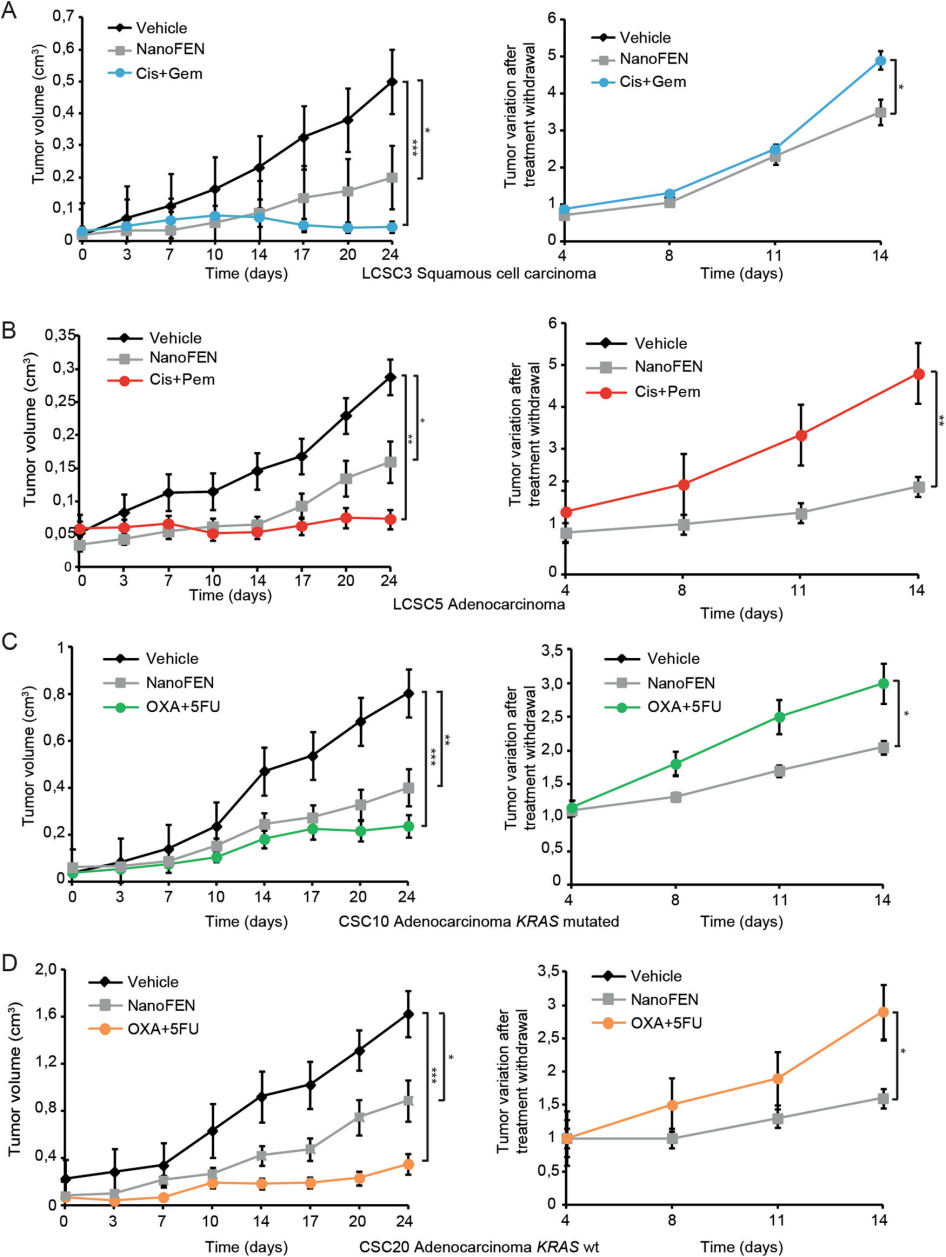
Antitumor efficacy of NanoFEN on lung and CRC xenografts. **a** Left panel, volume of xenografts derived from squamous cell carcinoma lung cancer spheroid cell line 3 (LCSC3) treated with vehicle (black diamonds), NanoFEN (grey squares) and cisplatin (Cis) plus gemcitabine (Gem) (blue circles); right panel, tumor variation after treatment withdrawal. **b** Left panel, volume of xenografts derived from adenocarcinoma lung cancer spheroid cell line 5 (LCSC5) treated with vehicle (black diamonds), NanoFEN (grey squares) and Cis plus pemetrexed (Pem) (red circles); right panel, tumor variation after treatment withdrawal. **c** Left panel, volume of xenografts derived from adenocarcinoma colorectal spheroid cell line 10 (CSC10) *KRAS* mutated treated with vehicle (black diamonds), NanoFEN (grey squares) and oxaliplatin (OXA) plus 5-fluorouracil (5FU) (green circles); right panel, tumor variation after treatment withdrawal. **d** Left panel, volume of xenografts derived from adenocarcinoma colorectal spheroid cell line 20 (CSC20) *KRAS* wild type, treated with vehicle (black diamonds), NanoFEN (grey squares) and OXA plus 5FU (orange circles); right panel, tumor variation after treatment withdrawal. Values represent the mean ± SEM of three independent experiments. *P < 0.05; **P < 0.01; ***P < 0.001 from one-way ANOVA and Bonferroni post-test

We assessed the antitumor effects of NanoFEN compared to conventional chemotherapeutic agents on lung and CRC xenografts derived from four primary spheroid lines by monitoring tumor growth during treatment and the variation of tumor size after treatment withdrawal. NanoFEN and chemotherapy treatment significantly inhibited tumor progression in lung squamous carcinoma xenografts, lung adenocarcinoma xenografts (Fig. 3a, b left) and CRC xenografts, the latter with or without mutated *KRAS* (Fig. 3c, d left). After treatment interruption, xenografts were monitored for two additional weeks in order to monitor the rate of tumor regrowth. While xenografts treated with chemotherapy showed accelerated growth rates after treatment cessation, NanoFEN-treated xenografts grew significantly slower, suggesting that NanoFEN does not induce an enrichment of tumorigenic cells (Fig. 3a–d right). Finally, we investigated the feasibility of a prolonged treatment with NanoFEN in mice. Mice bearing lung cancer xenografts were treated with NanoFEN for 70 days, during which we observed an extended arrest of tumor growth without any animal death or evident signs of toxicity (Supplementary Fig. 3a). Such observations, if confirmed in humans, could candidate NanoFEN as a compound suitable for long-term treatments such as those aimed at preventing tumor recurrence.

### Targeted phospho-proteomics on NanoFEN-treated primary cancer cells

**Fig. 4:**
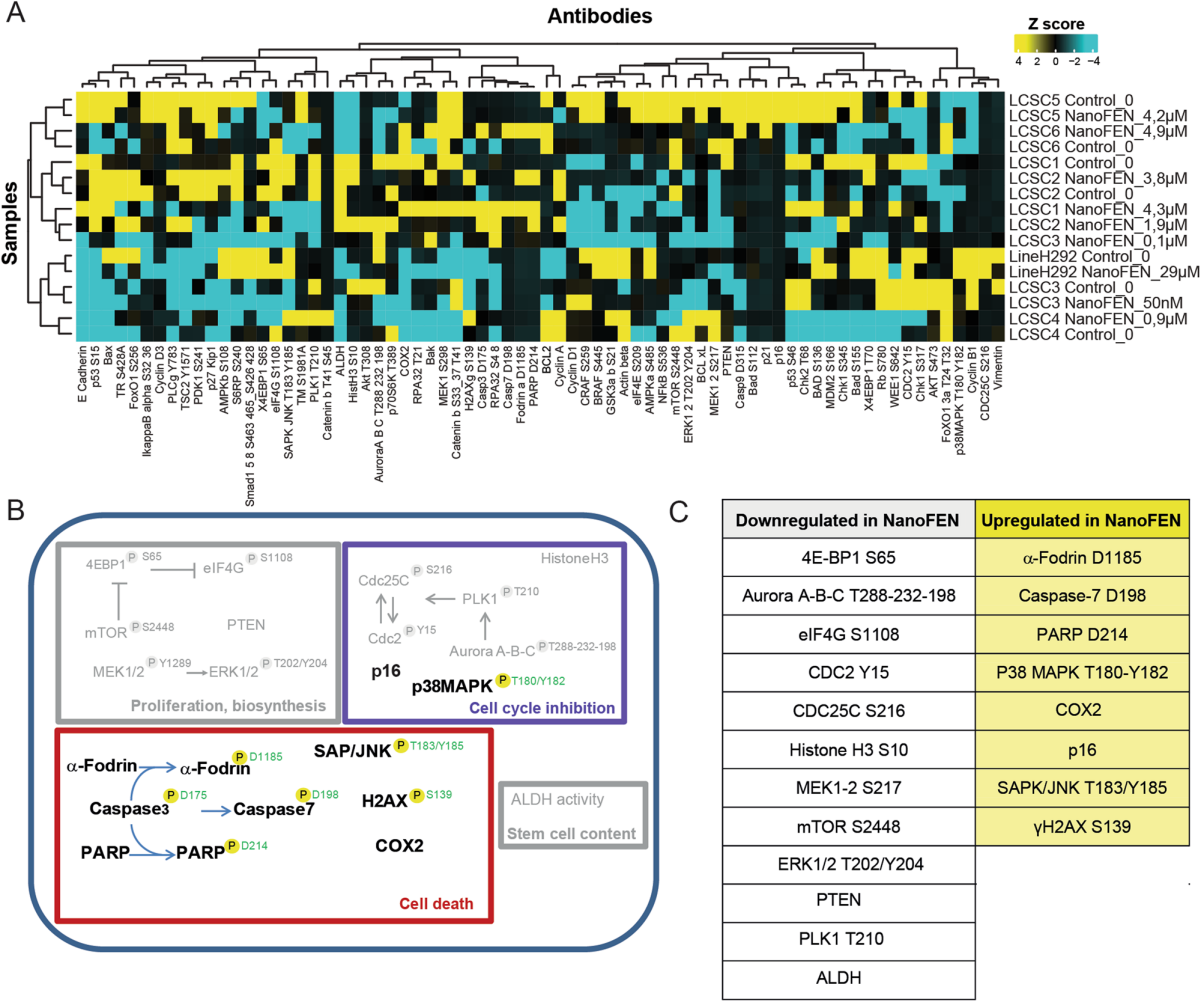
RPPA analysis of pathways activated by NanoFEN in primary lung cancer spheroid cells (LCSCs). **a** Hierarchical clustering of RPPA results obtained on six LCSCs derived from different histotypes and one commercial lung cancer cell line (H292) treated with the respective dose of NanoFEN for 48 h: LCSC1 4.3 μM; LCSC2 3.8 μM and 1.9 μM; LCSC3 0.1μM and 50 nM; LCSC4 0.9 μM; LCSC5 4.2 μM; LCSC6 4.9 μM; H292 29 μM. The color-coded values (high = yellow, average = black, cyan = low) in the heatmap correspond to normalized intensities of averaged sample replicates (*n* = 3), standardized over the sample set analyzed (*z* score). A list of RPPA antibodies used here is reported in Supplementary Table 1. **b** Graphical representation of the activated (colored) and repressed (grey) pathways emerged from RPPA analysis in LCSCs treated as described in (**a**). Phosphorylation sites are outlined in green when they result in protein activation. **c** Table highlights the endpoints upregulated in control (white panel) and treated (yellow panel) lung cancer cells as described in (**a**)

We aimed to generate a comprehensive picture of NanoFEN-induced changes in pathway activation by Reverse-Phase Protein Array (RPPA), a high-throughput technology based on the detection of proteins along with their post-traslational protein modifications, e.g., cleavage and phosphorylation^27^. To this end, we performed RPPA using a selection of 72 antibodies (Supplementary Table 1) on lung cancer spheroids left untreated or treated with NanoFEN at the IC50 dose. RPPA analysis revealed a NanoFEN-dependent modulation of multiple pathways involved in cell proliferation and biosynthesis, cell cycle inhibition, cell death and stemness. In detail, treatment with NanoFEN was associated with down-modulation of multiple components of the mTOR pathway, i.e., decreased levels of phospho-mTOR, phospho-eIF4G and phospho-4EBP1 or proliferation pathway, i.e., decreased levels of phospho-ERK 1/2; induction of cell death and DNA damage response, i.e., increased amounts of cleaved Caspase 7, cleaved PARP and phospho-H2A.Xγ and a decrease of cell cycle-related factors, i.e., phospho-Aurora A/B/C, phospho-CDC2, phospho-PLK1, phospho-CDC25c, phospho-Histone H3 as well as increased levels of p16 and of phospho-p38 MAPK, accompanied by a decrease of the stemness-related factor ALDH (Fig. 4a–c). Altogether, these results indicate that NanoFEN affects multiple cellular functions, whose readout endpoints underwent individual validation, as detailed in the following sections.

### Cell cycle and stemness inhibition by NanoFEN in lung and colorectal cancer spheroid cultures

**Fig. 5:**
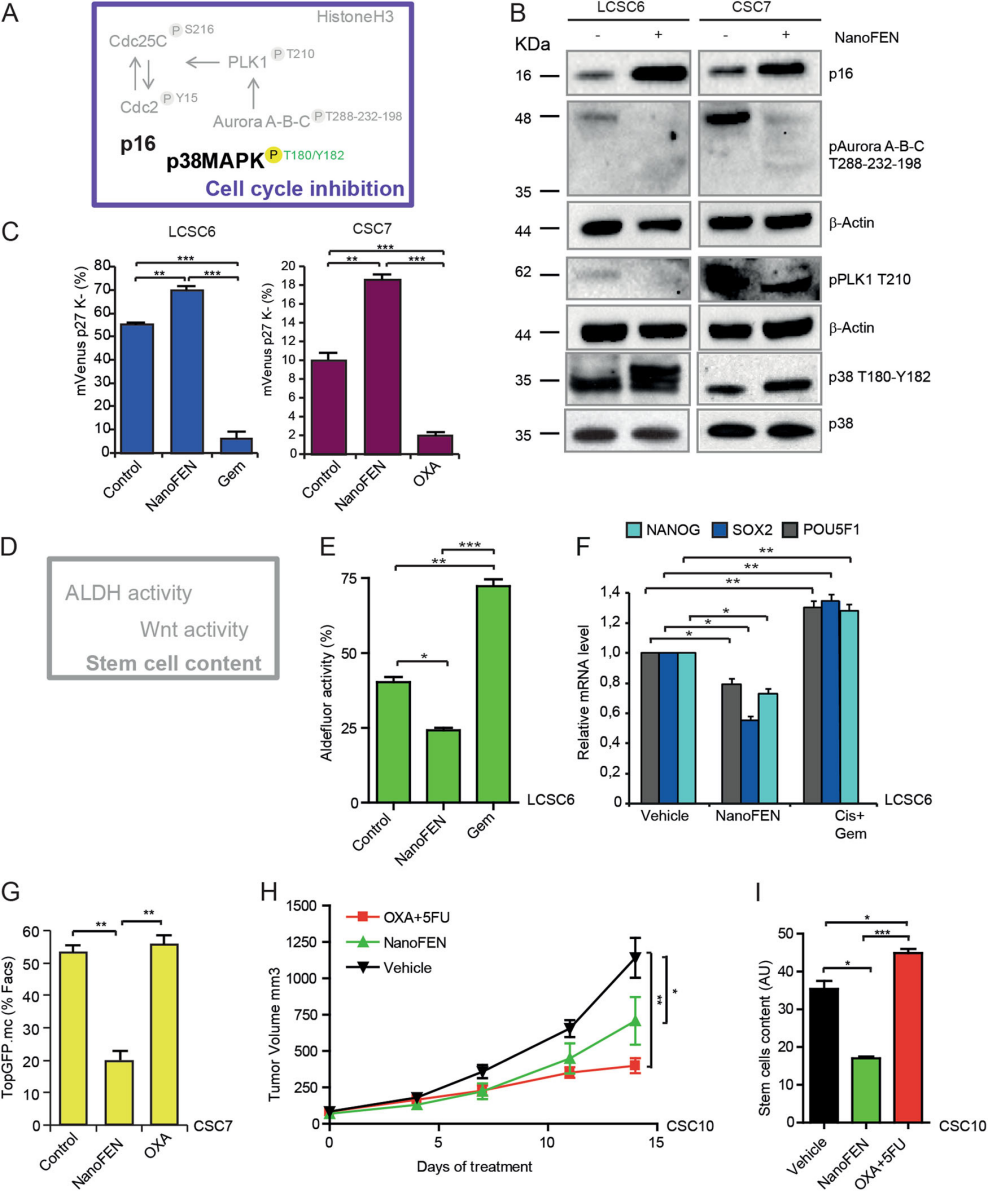
Effects of NanoFEN on cell cycle and stem-like features *in vitro* and *in vivo* on lung cancer spheroid cells (LCSCs) and CRC spheroid cells (CSCs). **a** Schematic representation of cell cycle regulators activated (black) or repressed (grey) arised from RPPA analysis of LCSCs treated with NanoFEN as described in Fig. 4a. **b** Immunoblot analysis of cell cycle regulators phospho-Aurora A-B-C, phospho-PLK1, p38, phospho-p38 and p16 on LCSC6 (left panel) and CSC7 (right panel) untreated and treated with NanoFEN at the IC50 concentration (4.9 μM and 0.2 μM respectively) for 48 h. **c** Flow cytometry analysis of mVenus p27K^−^-transduced LCSC6 (left panel) and CSC7 (right panel) untreated and treated with NanoFEN at the IC50 dose (4,9 μM and 0,2 μM respectively) for 48 h, Gem 25 μM and OXA 10 μM. Values represent mean ± SD of three independent experiments. **P < 0.01; ***P < 0.001 from two-tailed *t*-test. **d** Schematic representation of stem-like factors (repressed in grey) emerged from RPPA analysis of lung CSCs treated as described in Fig. 4a. **e** Cytofluorimetric analysis of Aldefluor activity on LCSC6 treated with NanoFEN at IC50 dose and Gemcytabine 25 μM for 48 h. Values represent mean ± SD of three independent experiments. **P < 0.01; ***P < 0.001 from two-tailed *t*-test. **f** qRT-PCR analysis of indicated genes on whole tumor lysates derived from LCSC6 xenografts, untreated or treated with NanoFEN 30 mg/Kg/week and Cis 3 mg/Kg/week and Gem 60 mg/Kg/week (Cis + Gem). Values represent mean ± SD of three independent experiments. *P < 0.05; **P < 0.01 from two-tailed *t*-test. **g** Flow cytometry analysis of TOP-GFP.mc-transduced CSC7, untreated and treated with NanoFEN at the IC50 dose 0.2 μM and OXA 10 μM for 48 h. Values represent mean ± SD of three independent experiments. **P < 0.01 from two-tailed *t*-test. **h** Volume of xenografts derived from CSC10 transduced with TOP-GFP.mc untreated (black downward triangles) or treated with vehicle or treated NanoFEN 30 mg/Kg/week (green upward triangles) and OXA 5 mg/Kg/week plus 5FU 12.5 mg/Kg/week (red squares). Values represent mean ± SEM three independent experiments. *P < 0.05; **P < 0.01 from one-way ANOVA and Bonferroni post-test. **i** Total stem cell content in tumor xenografts obtained as in (**h**), relative to the experiment shown in Supplementary Fig. 3c. Values were calculated as tumor weight × percentage of TOP-GFP.mc positive cells/100. Values represent mean ± SD of three independent experiments. *P < 0.05; ***P < 0.001 from two-tailed *t*-test

RPPA results indicated that NanoFEN modulated cell cycle-related factors (Fig. 5a), potentially resulting in growth slowdown and/or arrest. We confirmed the effects of NanoFEN on cell cycle factors by validating the increase in total p16 levels and phospho-p38 MAPK, and the decrease in phospho-Aurora A-B-C and phospho-PLK1 by immunoblotting in NanoFEN-treated lung and CRC spheroids (Fig. 5b). Then, we investigated whether NanoFEN was able to induce growth arrest through the use of the mVenus p27K^−^ construct, which identifies with yellow-green fluorescence the cells in G0 phase of the cell cycle and in G0/G1 transition^28^. Lung and colorectal spheroids were transduced with mVenus p27K^−^, treated with NanoFEN and evaluated by flow cytometry. Upon a 48 h treatment, NanoFEN significantly increased the number of Venus^+^ cells as compared with control cells indicating a higher number of G0 and G0/G1 transition cells, whereas chemotherapy strongly decreased Venus levels (Fig. 5c), indicating that in these conditions the two treatments have opposite effects of on the cell cycle. The decrease in ALDH levels observed in RPPA experiments prompted us to investigate whether NanoFEN would decrease tumor stem cell content (Fig. 5d). Aldefluor activity increased with chemotherapy, according to several studies showing that chemotherapeutic agents induce a population of drug-tolerant stem cells^29^, but decreased in NanoFEN-treated lung spheroids (Fig. 5e and Supplementary Fig. 3b). In line with this observation, we detected a decrease of genes related to stemness and self-renewal (*NANOG*, *SOX2*, *POU5F1*) upon NanoFEN treatment and a parallel increase in chemotherapy-treated cells (Fig. 5f) in lung spheroids. The TOP-GFP system provides a functional evaluation of stem cell content in CRC by recapitulating the expression of the Wnt target TCF linked to GFP^30^. Thus, we investigated TOP-GFP expression in vitro and in vivo upon NanoFEN treatment in cells transduced with a TOP-GFP.mCherry vector. NanoFEN significantly reduced TOP-GFP expression in vitro upon a short-term treatment (Fig. 5g). In vivo treatment with chemotherapy or NanoFEN of xenografts obtained with TOP-GFP-transduced CRC cells resulted, as expected, in decreased tumor growth as compared to controls (Fig. 5h). Importantly, analysis of TOP-GFP content at the end of the treatment in relation to tumor volume showed that the proportion of TOP-GFP^+^ cells increased upon chemotherapy but decreased upon NanoFEN treatment (Fig. 5i and Supplementary Fig. 3c). Altogether, these observations indicate that NanoFEN is able to induce a quiescent state and to decrease the content of cells with stem-like features in lung and CRC.

### Cell death pathways activated by NanoFEN in primary lung and CRC spheroid cells

**Fig. 6:**
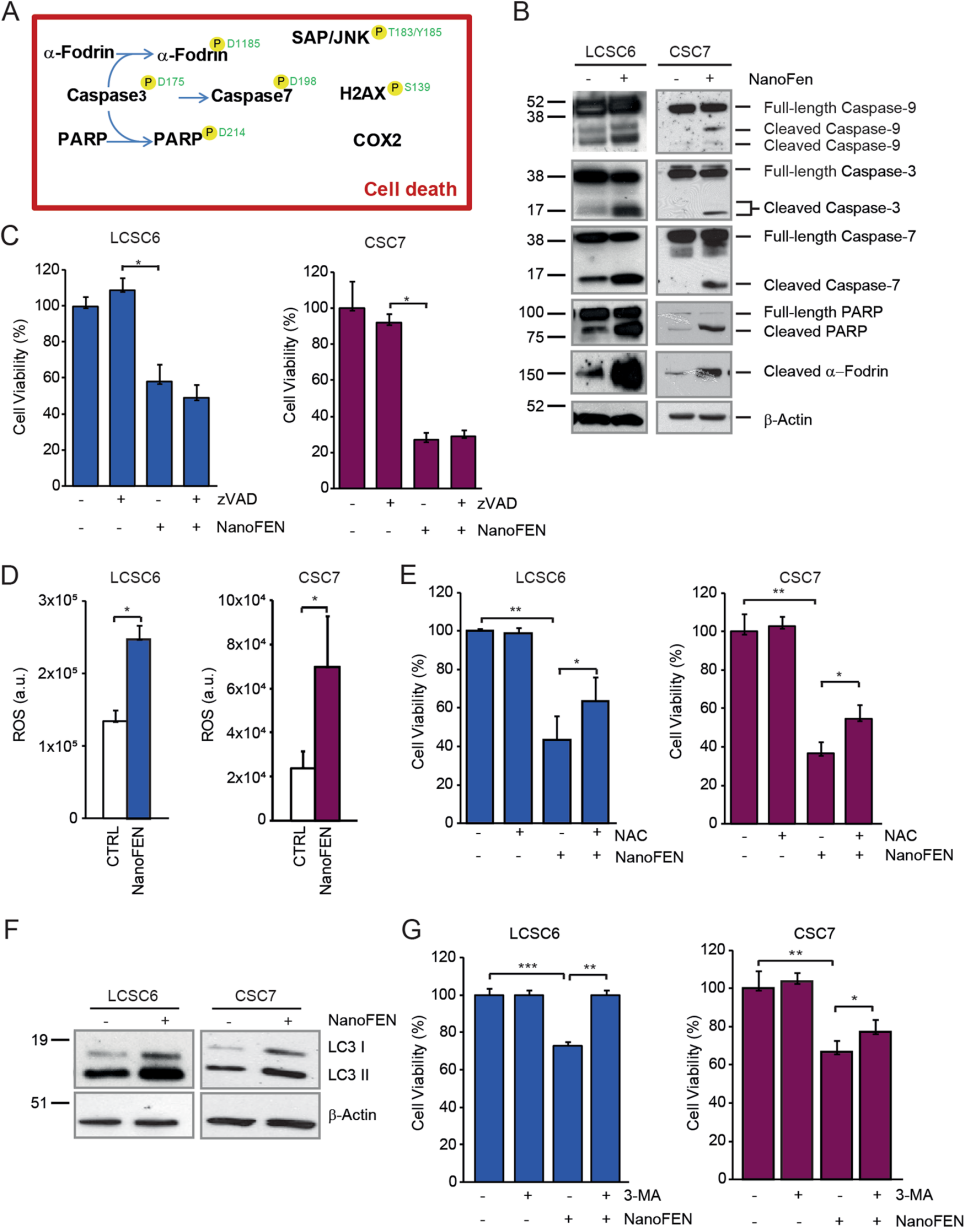
Analysis of cell death pathways activated by NanoFEN in lung cancer spheroid cells (LCSCs) and CRC spheroid cells (CSCs). **a** Schematic representation of RPPA endpoints involved in DNA damage, cell stress and apoptosis activated (black) in LCSCs treated with NanoFEN as described in Fig. 4a. Phosphorylation sites are outlined in green when they result in protein activation. **b** Immunoblot analysis of cell death-related proteins Caspase-9, 3, and 7, PARP and α-fodrin on LCSC6 (left panel) and CSC7 (right panel) untreated and treated with NanoFEN at IC50 dose (4.9 and 0.2 μM, respectively) for 48 h. **c** Cell viability of LCSC6 (left panel) and colon CSC7 (right panel) pretreated with zVAD 40 μΜ and then with NanoFEN at IC50 dose (4.9 and 0.2 μM, respectively) for 48 h. Values represent mean ± SD of three technical replicates of a representative experiment of two biological replicates. *P < 0.05 from two-tailed *t*-test. **d** Production of reactive oxygen species (ROS) was measured by flow cytometer in LCSC6 (left panel) and CSC7 (right panel) untreated or treated with NanoFEN (4.9 and 0.2 μM, respectively) for 24 h and then with 5 μM of CM-H_2_DCFDA. Values represent mean ± SD of three independent experiments. *P < 0.05 from two-tailed *t*-test. **e** Cell viability of LCSC6 (left panel) and CSC7 (right panel) pretreated with N-acetyl-cysteine (NAC) 2 mM for 1 h and after with NanoFEN for 48 h (4.9 and 0.2 μM respectively). Values represent mean ± SD of three technical replicates of a representative experiment. *P < 0.05; **P < 0.01 from two-tailed *t*-test. **f** Immunoblot analysis of autophagy-related marker LC3 I-II on LCSC6 (left panel) and CSC7 (right panel) untreated and treated with NanoFEN at IC50 dose (4.9 and 0.2 μM, respectively) for 24 h. **g** Cell viability of LCSC6 (left panel) and CSC7 (right panel) treated with 3-Methyladenine (3-MA) 5 mM plus NanoFEN (4.9 and 0.2 μM, respectively) for 24 h. Values represent mean ± SD of a three technical replicates of a representative experiment. *P < 0.05; **P < 0.01; ***P < 0.001 from two-tailed *t*-test

As shown by RPPA analyses, NanoFEN treatment affects several factors involved in DNA damage, cellular stress and apoptosis. To gain further insights into death-related events activated by NanoFEN we validated and extended RPPA results by immunoblotting in lung and colon spheroids, thus confirming NanoFEN-mediated caspase activation and cleavage of apoptotic substrates (Fig. 6a, b and Supplementary Fig. 4a). Pre-treatment with the pan-caspase inhibitor zVAD-fmk (zVAD) did not substantially inhibit NanoFEN-induced toxicity, suggesting that caspase activity is dispensable for NanoFEN-induced death (Fig. 6c), similarly to what has been described for the classical formulation of fenretinide. Because reactive oxygen species (ROS) generation has been reported to occur upon fenretinide treatment in different cellular systems^31^, we next investigated the production of ROS in NanoFEN-treated lung and colon spheroids. We detected a moderate increase in ROS levels in both lung and CRC cells, as shown by H_2_-DCFDA staining (Fig. 6d). The ROS scavenger N-acetyl-cysteine (NAC) partially rescued NanoFEN-induced death in both lung and colon CSCs and reduced ROS levels (Fig. 6e and Supplementary Fig. 4b) suggesting that ROS generation plays a role in NanoFEN cytotoxicity, in line to what reported in leukemic stem cells^32^. Since the repression of the mTOR pathway that emerged from RPPA analyses is known to promote autophagy, we investigated the presence of autophagy-related markers in lung and CRC spheroids treated in vitro with NanoFEN. Interestingly, we found in the majority (3 out of 4) of the spheroid lines tested an increased expression of both LC3 and its phosphatidylethanolamine conjugate (LC3-II), which is recruited to autophagosomal membranes^33^ (Fig. 6f and Supplementary Fig. 4c). We also observed that the autophagy inhibitor 3-MA was able to counteract NanoFEN-induced death (Fig. 6g), suggesting that autophagy contributes to NanoFEN-induced death in both lung and CRC cells.

### Modulation of cellular metabolism by NanoFEN

**Fig. 7:**
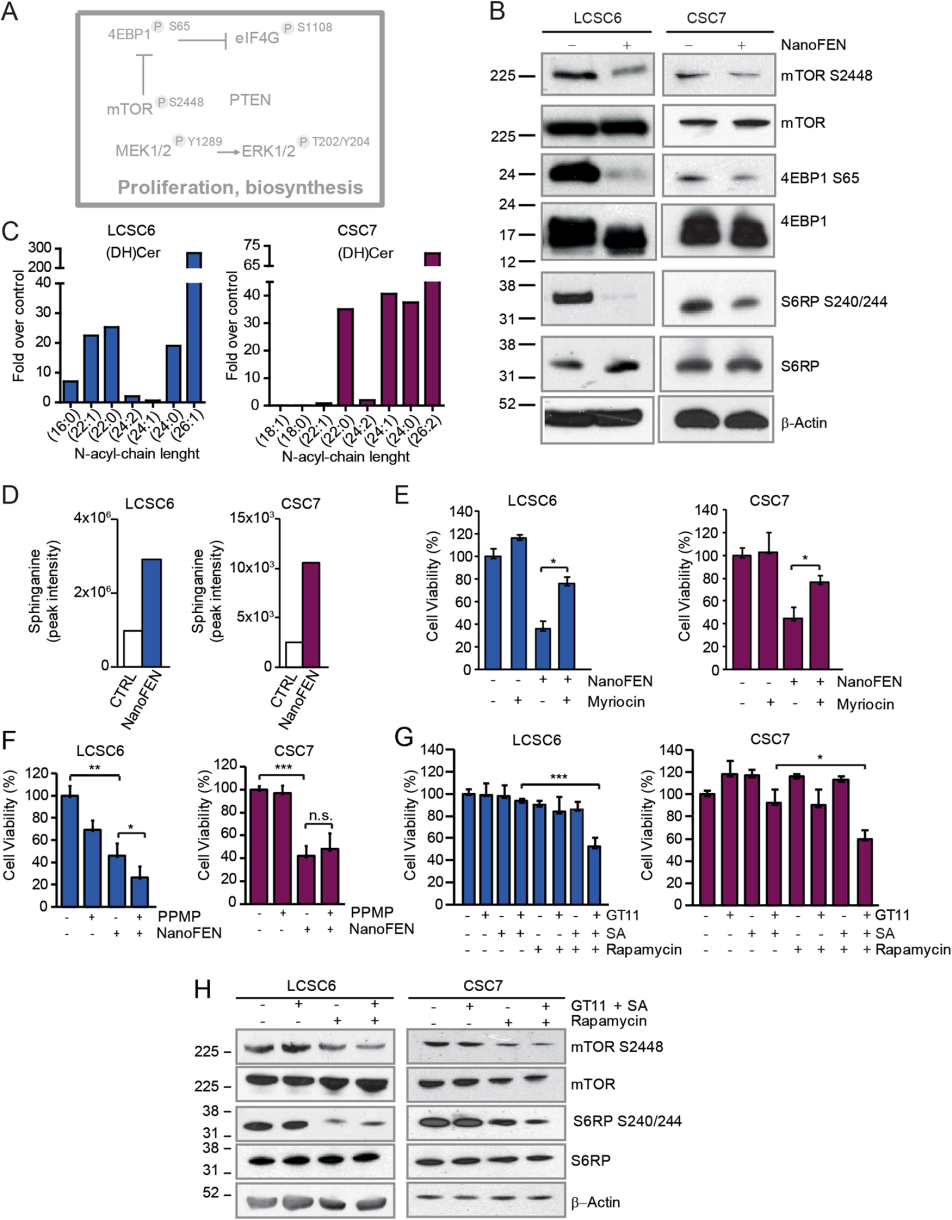
NanoFEN inhibits cellular metabolism in lung cancer spheroid cells (LCSCs) and CRC spheroid cells (CSCs). **a** Schematic representation of RPPA results of proliferative and biosynthetic pathways repressed (grey) in LCSCs treated with NanoFEN. **b** Immunoblot analysis of proteins involved in metabolic process on LCSC6 (left panel) and CSC7 (right panel) untreated and treated with NanoFEN at IC50 dose (4.9 and 0.2 μM, respectively) for 48 h. **c** Graphs represent NanoFEN effects on lipid metabolism by liquid chromatography-mass spectrometry (LC-MS) on LCSC6 (left panel) and CSC7 (right panel). Values represents the fold over control of treated cells with NanoFEN (4.9 and 0.2 μM, respectively) for 24 h. **d** Sphinganine peak intensity in LCSC6 (left panel) and CSC7 (right panel) treated with NanoFEN (4.9 and 0.2 μM, respectively) for 24 h. **e** Cell viability of LCSC6 (left panel) and CSC7 (right panel) treated with Myriocin 1 μM plus NanoFEN (4.9 and 0.2 μM, respectively) for 48 h. Values represent mean ± SD of three independent experiments. *P < 0.05, from two-tailed *t*-test. **f** Cell viability of LCSC6 (left panel) and CSC7 (right panel) treated with glucosylceramide synthase inhibitor PPMP 10 μM plus NanoFEN (4.9 and 0.2 μM, respectively) for 48 h. Values represent mean ± SD of three independent experiments. *P < 0.05, from two-tailed *t*-test. **g** Cell viability of LCSC6 (left panel) and CSC7 (right panel) treated with dihydroceramide desaturase inhibitor (GT11) 0.5 μM plus sphinganine (SA) 2 μM plus mTOR pathway inhibitor Rapamycin 10 nM for 48 h. Values represent mean ± SD of three independent experiments. *P < 0.05; ***P < 0.001, from two-tailed *t*-test. **h** Immunoblot analysis of mTOR pathway components mTOR, phospho-mTOR, S6RP and phospho-S6RP on LCSC6 (left panel) and CSC7 (right panel) treated as in (**g**)

Fenretinide treatment of cancer cells has been previously associated with the production of lipid second messengers including ceramide, gangliosides (both involved in apoptosis induction) and dihydroceramide^34,35^. Moreover, RPPA results showed a global inhibition of the mTOR pathway (Fig. 7a) that was subsequently confirmed by immunoblotting (Fig. 7b and Supplementary Fig. 5a), thus linking NanoFEN mechanism of action to metabolism, biosynthesis and lipid signaling networks. We assessed the effect of NanoFEN on cellular lipids by liquid chromatography-mass spectrometry (LC-MS) in lung and CRC spheroid cultures, revealing a massive accumulation of d18 dihydroceramide in treated cells (Fig. 7c and Supplementary Fig. 5b) as compared to other ceramides (Supplementary Fig. 5c–g). Dihydroceramide production was previously shown to elicit multiple biological effects in cancer cells such as G0/G1 cell cycle arrest, impairment of mitochondrial function and autophagy induction^36–38^, corresponding to the biological effects observed in NanoFEN-treated cells. These observations suggest that NanoFEN (as was previously shown for fenretinide) targets dihydroceramide desaturase (DES), thus inducing dihydroceramide accumulation. Accordingly, we observed a strong increase of sphinganine (SA, the lipid mediator upstream of DES) both in vitro in NanoFEN-treated lung and CRC cells (Fig. 7d) and in vivo in lung cancer xenografts (Supplementary Fig. 6a). In order to determine the functional relevance of dihydroceramide production in NanoFEN-treated cells we used the serine palmitoyltransferase inhibitor myriocin, which blocks de novo sphingolipid biosynthesis^39^. Pretreatment of spheroid lines with myriocin partially protected cells from NanoFEN-induced death, indicating an active role of the sphingolipid pathway in mediating NanoFEN effects (Fig. 7e). In line with an important role of dihydroceramide in mediating NanoFEN effects, SA and the DES inhibitor GT11 induced a dose-dependent viability decrease in lung cancer spheroids (Supplementary Fig. 6b). By contrast, the glucosylceramide synthase inhibitor 1-phenyl-2-palmitoylamino-3-morpholino-1-propanol (PPMP) was unable to block NanoFEN-induced death, indicating that the cytotoxic effects of NanoFEN are determined by the accumulation of dihydroceramide rather than by glucosylceramide/ceramide formation (Fig. 7f). Finally, we ought to determine whether the sphingolipid pathway would cooperate with repression of the mTOR pathway in mediating NanoFEN-induced toxicity. To this aim, we mimicked the effect of NanoFEN by treating spheroids with both sphinganine and GT11 at low doses. Additional treatment with the mTOR pathway inhibitor rapamycin significantly potentiated the toxic effects of the SA/GT11 combination, indicating a cooperative effect of the two pathways in inducing cancer cell death (Fig. 7g and Supplementary Fig. 6c). Western blot analysis of key factors implicated in mTOR signaling showed that the combined treatment with SA/GT11/rapamycin effectively inhibited the mTOR pathway and recapitulated NanoFEN-related events (Fig. 7h).

## Discussion

In accordance to their multifaceted role in cell proliferation, differentiation and death, retinoids have long been studied for their chemopreventive and chemotherapeutic effects in cancer patients, although their mechanism of action has not been completely elucidated. One of the most promising retinoid derivatives is fenretinide, which has been extensively investigated in phase I-III clinical trials, revealing long-term safety and tolerability but modest anticancer effects^3,4,7–15,21,40–43^. The scarce efficacy of fenretinide in previous clinical studies is related to its low bioavailability, as even high-dose schedules and multiple administrations did not provide plasma concentrations sustained over time at the levels required for a therapeutic activity^3,8,10–13^. We report here that a nanoencapsulated form of fenretinide (previously modified through a salification procedure in order to increase complexation effectiveness) has an enhanced solubility and reaches significantly higher plasma levels than formulations previously used in clinical trials. The mechanism of action of NanoFEN was analyzed in primary spheroid cultures of lung and CRC, thus providing a comprehensive picture of protein and lipid pathways activated in cancer cells. The results emerging from proteomic and lipidomic analyses revealed a complex set of molecular events activated by NanoFEN, resulting in a broad landscape of functional effects ranging from cell death (with features of both autophagy and apoptosis) to cell cycle arrest and to a generalized metabolic repression mediated by inhibition of the mTOR pathway and massive production of dihydroceramide. The effect of NanoFEN in inducing growth arrest/quiescence is particularly interesting in view of a potential use of this compound in chemopreventive settings. In fact, maintaining tumor cells (particularly those disseminated in distant organs) in a prolonged and stable quiescent state is progressively emerging as a potential therapeutic strategy aimed at preventing tumor relapse^44^. In line with this hypothesis, a combination of all-trans retinoic acid and 5-azacitidine has been reported to induce a quiescent state in bone marrow-disseminated cancer cells, supporting a role of retinoids in promoting a long-term metastasis-free condition^45–47^ and is currently being investigated in a clinical trial (NCT03572387). If confirmed in additional preclinical and clinical studies, the low toxicity of NanoFEN would allow prolonged treatment schedules aimed at avoiding the reactivation of disseminated tumor cells and subsequent tumor relapse, particularly in cancers subject to late recurrences such as those of breast and prostate. Moreover, it would be interesting to investigate the effect of NanoFEN in retinoid-sensitive tumors such as some hematological tumors as well as neuroblastoma, where the increased bioavailability of NanoFEN may allow more effective treatment schedules as compared to previous clinical trials. In summary, the results reported in this study indicate NanoFEN as a bioavailable fenretinide formulation with broad-range inhibitory effects on cancer cells that may find a future use in several clinical settings.

## Materials and methods

### Cells, tissues, mice, antibodies, and reagents

Colon and lung cancer specimens were obtained from patients undergoing surgical resection upon informed consent and approval by the Sapienza-Policlinico Umberto I Ethical Committee (RIF.CE: 410717/10/2016). Spheroid cultures were established and validated as previously described^48,49^. All the commercial cancer cell line were purchased from the American Type Culture Collection (ATCC, Manassas, VA, USA) or Sigma-Aldrich (St. Louis, MO, USA) and cultured following manufacturer’s instructions. Fenretinide, N-(4-Hydroxyphenyl) retinamide (code 65646-68-6) was purchased from Olon (Milan, Italy). 2-hydroxypropyl-β-cyclodextrin (average Mw ~1380), potassium hydroxide, 1-octanol, D_2_O, DMSO-d6 were purchased from Sigma-Aldrich. Antibodies used for RPPA and western blot were listed in Supplementary Table 1. Oxaliplatin and 5-fluorouracil were from Teva Italia (Milano, Italy) gemcitabine and cisplatin were from Selleckchem (Houston, TX, USA). Aldefluor assay was from STEMCELL Technologies (Vancouver, Canada). Myriocin was purchased from ENZO Life Science (New York, NY, USA), N-[(1R,2S)-2-hydroxy-1-hydroxymethyl-2-(2-tridecyl-1-cyclopropenyl)ethyl]octanamide (GT11) and Sphinganine from Avanti Polar Lipids (Alabaster, AL, USA), Rapamycin from Selleckchem, z-Val-Ala-DL-Asp-fluoromethylketone (zVAD) from BACHEM (Torrance, CA, USA), DL-threo-1-Phenyl-2-hexadecanoylamino-3-morpholino-propanol-HCL (PPMP) from Matreya (State College, PA, USA), N-Acetyl-L-Cysteine (NAC) was purchased from Sigma-Aldrich, 5-chloromethylfluoresceindiacetate (CMFDA) was purchased from Molecular Probes (Eugene, OR, USA). Anti-LC3 and anti-β-Actin antibodies were purchased from Sigma-Aldrich; phospho-Aurora A (Thr288)/Aurora B (Thr232)/Aurora C (Thr198) (D13A11), phospho-PLK1, phospho-S6 ribosomal protein (Ser240/244) and total S6 ribosomal protein (5G10), caspase3, caspase7, caspase9, PARP, fodrin cleaved, phospho-p38 MAP kinase (Thr180/Tyr182), mTOR, phospho-mTOR (Ser2448), 4E-BP1(53H11), phospho-4E-BP1(Ser65) antibodies were from Cell Signaling Technology (Danvers, MA, USA). Anti-CDKN2A (p16) was from Biolegend (London, United Kingdom). Secondary anti-mouse and anti-rabbit antibodies coupled to horseradish peroxidase were from Bio-Rad (Hercules, CA, USA).

### Preparation of fenretinide 2-hydroxypropyl-β-cyclodextrin complex (NanoFEN)

The first step of NanoFEN preparation involved fenretinide salification to raise its aqueous solubility to the extent required for complexation with cyclodextrins. Salification was carried out by dissolving fenretinide (2 mol in 20 mL ethanol containing a stoichiometric amount of potassium hydroxide. The solvent was removed in a rotary evaporator to obtain a solid red residue of fenretinide potassium salt (4-HPRK). The salt was physically mixed with 2-hydroxypropyl-β-cyclodextrin at 1:10 w:wsalt:cyclodetrin ratio and grinded to homogeneity. The physical mixture obtained was dispersed in 30 mL water, stirred at room temperature 6 h in the dark and subsequently left at 4 °C overnight. After this period the suspension was stirred again for 2 h at room temperature, diluted with 20 mL water and finally filtered through a 0.2 µm filter to eliminate any excess of undissolved material. The filtered solution was lyophilized until the solid complex was obtained as an orange-red homogeneous powder. The complex was analyzed by ^1^H-NMR to detect the presence of chemical shift changes with respect to the free drug as indicators of fenretinide inclusion into the cyclodextrin hydrophobic cavity^50^. For the analysis the complex was dissolved in D_2_O and the fenretinide salt in DMSO-d6. The ^1^H-NMR spectra were recorded by an Inova 600 MHz High Resolution NMR Spectrometer. The amount of fenretinide incorporated into the complex was spectrophotometrically determined in solutions obtained by dissolving known amounts of the solid complex in a water:ethanol (1:1, v-v) mixture with the aim to dissociate the drug from the complex and maintain its solubilization in the aqueous phase in the presence of ethanol. The hydro-alcoholic solutions were analyzed for their fenretinide content at the maximum wavelength of the fenretinide absorption (360 nm). The aqueous solubility of fenretinide complexed with 2-hydroxypropyl-β-cyclodextrin was measured in comparison with the pure drug. Excess amounts of the complex or pure drug were placed in water, stirred 12 h at 25 °C in the dark to saturation of the aqueous phase and filtered through 0.2 µm filters. After filtration the saturated solutions were spectrophotometrically analysed at 360 nm for their fenretinide content.

### Release studies

Release studies of fenretinide from the complex were carried out by placing saturated aqueous solutions of the complex in a dialysis membrane bag (Mw cutoff 5KD), allowing diffusion only to the free drug and not to the complex. The membrane separated the saturated aqueous solution of the complex from a receiving compartment containing an aqueous buffer (PBS pH 7.4) and 1-octanol (10:1v:v ratio) used to extract the drug diffused through the dialysis membrane and to simulate the presence of hydrophobic absorbing phases such as biomembranes^51^. The release of the drug from the complex was evaluated by a spectrophotometric analysis of the complex solution in the releasing compartment at the maximum absorption wavelength of fenretinide (360 nm) at appropriate time points.

### Pharmacokinetics studies

The experiments were performed in CD1 female mice, 7 weeks of age, (body weight 25 ± 2 g) obtained from Envigo RMS SrL (Udine, Italy). Mice were maintained under specific-pathogen-free conditions with constant temperature and humidity, according to the institutional guidelines of the Istituto Superiore di Sanità Animal Care Committee. Animal experimentation was conducted in conformance with the following laws, regulations, and policies governing the care and use of laboratory animals: Italian Governing Law (D. l. 26/2014; Authorization n.19/2008-A issued March 6, 2008 by the Ministry of Health). The pharmacokinetics of NanoFEN, administered by oral or intravenous route, was investigated in mice receiving the formulation at doses corresponding to 5 mg/kg of fenretinide. The intravenous administration was carried out by injection in the tail vein of an intravenous bolus of NanoFEN dissolved in water or PureFEN suspended in a mixture of ethanol 10 % in saline as a comparison. For the oral administration the mice were gavaged the with NanoFEN dissolved in water or with the reconstituted content of the NCI capules, i.e., fenretinide (100 mg) in corn oil (704 mg) and Polysorbate 80 (60 mg) as a comparison. After the intravenous or oral treatments, a series of blood samples were taken at 15 and 30 min and at 1, 2, 4, 8, 10, 24 and 48 h. Blood was collected in heparinized tubes from the retro-orbital plexus of the mice under isoflurane anesthesia. To obtain plasma, blood samples were centrifuged at 4000 rpm for 10 min at 4 °C. The samples were stored at −20 °C until analysis. The total concentration of fenretinide in plasma of mice was determined according to a method previously described^52^. Briefly, aliquot of 200 μl of each plasma sample was added to 400 μl of Acetonitrile (CH_3_CN) containing 125 μg/mL Butylated hydroxytoluene (BHT), and the mixture was vortexed and centrifuged to pellet the precipitated proteins. The recovered supernatants were analysed on a liquid chromatograph (Perkin-Elmer, Norwall, CT) fitted with a C18 (50 mm × 2.0 mm, 5 μm) reverse-phase column and a C18 precolumn (Perkin-Elmer, Milan, Italy). The mobile phase consisted of CH_3_CN:H2O:CH_3_COOH (75:2:2, v:v:v) delivered at a flow rate of 2 mL/min. Detection was carried out with a Perkin-Elmer LC95 absorbance detector at 362 nm. N-(4-ethoxyphenyl)-retinamide (EPR) was used as internal standard. For the quantitative evaluation, reference standard curves were set up in control mouse plasma with different known amounts of NanoFEN or PureFEN. The measured fenretinide concentration-vs-time data were elaborated by non-compartmental analysis (WinNonLin software v3) to obtain the main pharmacokinetic parameters of NanoFEN.

### Animal procedures

All animal procedures were performed according to the Italian national animal experimentation guidelines (D.L.116/92) upon approval of the experimental protocol by the Italian Ministry of Health’s Animal Experimentation Committee (DM n. 292/2015 PR 23/4/2015). 6-week-old female NOD.Cg-Prkdcscid Il2rgtm1Wjl/SzJ (NSG) mice were purchased from The Jackson Laboratory (Bar Harbor, Maine, USA) for colon and lung cancer xenografts. Tumors were measured twice weekly by an external digital caliper, and volumes were calculated using the following formula: π/6 x d2 x D, where d and D represent shorter and longer tumor measurements, respectively. Drug treatments started when tumor volume was 50-100 mm^3^: mice were randomized in control and treatment group and treated with NanoFEN 10 mg/Kg/threeweekly/intraperitoneally (IP), chemotherapeutic agent combinations as cisplatin 3 mg/kg/biweekly/IP+pemetrexed 200 mg/kg/biweekly/IP or cisplatin 3 mg/kg/biweekly/IP+gemcitabine 60 mg/kg/biweekly/IP or 5-fluorouracil 12,5 mg/Kg+oxaliplatin 5 mg/Kg/biweekly/IP. Control animals were treated with vehicle only. Tumors were measured at the indicated timepoints and mice were monitored for signs of drug-induced toxicity and weighed regularly. At the end of experiments, tumors were collected and dissociated to obtain cell suspension for subsequent studies or monitored to evaluate tumor growth rate after treatment interruption. Relative tumor growth is indicated as ratio of tumor volume at the indicated days after drug suspension versus volume at the last day of treatment. Animals were euthanized according to the national Animal Welfare Guidelines.

### Lentiviral infection

Primary spheroid cultures of colon and lung cancer cells were stably transduced with mVenus-p27K^−28^ or TOP-GFP.mCherry (purchased from Addgene, Cambridge, MA, USA) using ProFection® Mammalian Transfection System from Promega (Madison, Wisconsin, USA) following manufacturer’s instructions.

### Metabolite extraction

Samples were extracted following the protocol by D’Alessandro et al^53^. Samples were resuspended in 0.15 mL of ice cold ultra-pure water (18 MΩ) to lyse cells, then the tubes were plunged into a water bath at 37 °C for 0.5 min. Samples were mixed with 0.6 mL of −20 °C methanol and then with 0.45 mL chloroform. Subsequently, 0.15 mL of ice cold ultra-pure water were added to each tube and then transferred to −20 °C for 2–8 h. An equivalent volume of acetonitrile was added to the tube and transferred to 4 °C for 20 min. Samples were thus centrifuged at 10,000×*g* for 10 min at 4 °C. Finally, samples were dried in a rotational vacuum concentrator (RVC 2-18—Christ Gmbh; Osterode am Harz, Germany) and re-suspended in 200 µl of water, 5% formic acid and transfer to glass auto-sampler vials for LC/MS analysis.

### Rapid resolution reversed-phase HPLC

An Ultimate 3000 Rapid Resolution HPLC system (LC Packings, DIONEX, Sunnyvale, USA) was used to perform metabolite separation. The system featured a binary pump and vacuum degasser, well-plate autosampler with a six-port micro-switching valve, a thermostated column compartment. Samples were loaded onto a Reprosil C18 column (2.0 mm × 150 mm, 2.5 µm - Dr Maisch, Germany) for metabolite separation. For lipids multi-step gradient program was used. It started with 8% solvent A (ddH20, 20 mmol L-1 ammonium formiate; pH 5) to 6% solvent A for 3 min than to 2% solvent A for 35 min and finally to 100% solvent B (methanol) in 30 min. At the end of gradient, the column was reconditioned with 8% solvent A for 5 min.

### Mass spectrometry analysis through microTOF-Q

Mass spectrometry analysis was carried out on an electrospray hybrid quadrupole time of Flight mass spectrometer MicroTOF-Q (Bruker-Daltonik, Bremen, Germany) equipped with an ESI-ion source. MS analysis was carried out capillary voltage 2800 V, nebulizer 45psi and dry gas of 9 l/min, scan mode 100–1500 *m/z*. Data were acquired with a stored mass range of 100–2000 *m/z*. Tandem mass spectrometry (MS/MS) is used for lipid species structural characterization. Automatic isolation and fragmentation (AutoMSn mode) was performed on the four most intense ions simultaneously throughout the whole scanning period (30 min per run). Calibration of the mass analyzer is essential in order to maintain an high level of mass accuracy. Instrument calibration was performed externally every day with a sodium formate solution consisting of 10 mM sodium hydroxide in 50 % isopropanol, water, 0.1 % formic acid. Automated internal mass scale calibration was performed through direct automated injection of the calibration solution at the beginning and at the end of each run by a 6-port divert-valve.

### Real time PCR

RNA from vehicle and treated xenografts was extracted with TRIzol (Thermo Fisher Scientific, Waltham, Massachusetts, USA) following manufacturer’s instructions. 1 µg of RNA were reverse transcribed with M-MLV reverse transcriptase (Thermo Fisher Scientific) and 50 ng of cDNA were used as template in the PCR reactions. Specific probes used for *NANOG* (Hs04399610_g1), *SOX 2* (Hs04260357_g1) and *POU5F1* (Hs00999634_gH) were all from Thermo Fisher Scientific. Normalization was performed using β-actin (Hs00194899_m1) as reference. Values were expressed in terms of 2^−ΔΔCt^ where ΔΔCt = ΔCt sample−ΔCt calibrator. ΔCt is the difference in threshold cycles between the specific RNA target and reference gene amplicons given by StepOne Plus Real-Time PCR software by negative correlation with an internal reference dye (ROX).

### Reverse-phase protein array

For RPPA experiments, primary lung cancer spheroid cells (LCSCs) and commercial lung cancer cell lines were left untreated or treated with NanoFEN (IC50 concentration) as follows: LCSC1 (4.3 µM), LCSC 2 (1.9 and 3.8 µM) LCSC 3 (50 nM and 1 µM) LCSC 4 (0.9 µM) LCSC 5 (4.2 µM) LCSC 6 (4.9 µM) H292 (29 µM). Following 48 h treatment of the six LCSCs and H292 commercial cell line, cell pellets were promptly lysed in a buffer containing T-PER reagent (Thermo Fisher Scientific), 300 mM NaCl (J.T.Baker; Avantor Performance Materials, Center Valley, PA), protease and phosphatase inhibitors cocktails (Sigma-Aldrich) and stored at −80 °C. In order to prepare protein samples for RPPA printing, protein lysates were allowed to thaw on ice, their total protein concentration was measured using the Bradford reagent method (Thermo Fisher Scientific) and the volume corresponding to 50 μg of total protein was used to further dilute samples to a final concentration of 0.5 mg/mL, as follows: 50% 2X Tris-Glycine SDS Sample Buffer (Life Technologies Corporation, Carlsbad, CA), up to 47.5% sample volume and T-PER reagent (Thermo Fisher Scientific) and 2.5% Tris (2-carboxyethyl)phosphine hydrochloride (TCEP) reagent (Thermo Fisher Scientific). RPPA lysates were boiled for 3 min and stored at −80 °C until further processing. Prior to printing onto nitrocellulose-coated slides (GRACE Bio-Labs, Bend, OR) using a robotic arrayer (Aushon Biosystems, Billerica, MA, USA), RPPA lysates were allowed to thaw up to room temperature and boiled for an additional 3 min. All samples were printed in technical triplicate as neat and 1:4 dilution pairs. Reference standard lysates, i.e., HeLa+Pervanadate (Becton, Dickinson and Company, Franklin Lakes, NJ, USA), A431+EGF (Becton, Dickinson and Company), Jurkat+Etoposide (Cell Signaling Technology) and Jurkat+Calyculin A (Cell Signaling Technology), were printed in 10-point decreasing mixtures of treated to untreated samples as positive and quality controls of antibody staining. Each reference standard curve was printed in technical triplicates at a final concentration of 0.5 mg/mL. A selected subset of the printed microarray slides were stained with Sypro Ruby Protein Blot Stain (Thermo Fisher Scientific) to estimate sample total protein concentration and the remaining slides were stored under desiccated conditions at -20 °C. Immediately before antibody staining, printed slides were treated with 1X Reblot Mild Solution (Chemicon, Burlington, MA, USA) for 15 min, washed 2 × 5 min with PBS and incubated for 2 h in blocking solution containing 2% I-Block (Applied Biosystems, Foster, CA, USA) and 0.1% Tween-20 in PBS. Immunostaining was carried out using a tyramide-biotin signal amplification kit (DAKO, Santa Clara, CA, USA). Primary antibody binding was detected using a biotinylated goat anti-rabbit IgG H+L (diluted at 1:7500; Vector Laboratories, Burlingame, CA, USA) or rabbit anti-mouse Ig (diluted at 1:10, DAKO) followed by streptavidin-conjugated IRDye®-680LT fluorophore (LI-COR Biosciences, Lincoln, Nebraska, USA. Primary antibodies undergo pre- and post-RPPA validation for single band specificity by western-blot using complex cellular lysates. Negative control slides, incubated only with secondary antibody were included in each staining run. All Sypro Ruby, i.e., per-spot total protein content,as well as immunostained slides were scanned using a Tecan Power Scanner™ (Tecan Group Ltd, Männedorf, Switzerland) at 5 μm resolution. Acquired images were analyzed with MicroVigene v5.2, http://www.vigenetech.com/MicroVigene.htm (VigeneTech Inc, Carlisle, MA, USA), for spot detection, local and negative control background subtraction, replicate averaging and total protein normalization. RPPA data analysis was performed by means of ‘R’ v3.5.0 https://www.R-project.org/ (R Foundation for Statistical Computing) and ‘RStudio’ v1.1.414 https://www.rstudio.com/ (RStudio), using the following installed packages:base, plyr, tidyverse, FactoMineR, factoextra, RColorBrewer, Bioconductor and shiny. Custom ‘R’ scripts for slide quality control, internal standardization and two-way hierarchical clustering (correlation distance and complete method) are available upon request. A detailed list of antibodies used for RPPA is available in Supplementary Table 1.

### Flow cytometry

For flow cytometry analysis, primary lung and CRC spheroid cultures transduced with the mVenus p27K^−^ and treated with NanoFEN, oxaliplatin or gemcitabine were dissociated at a single cell level mechanically or with TrypLE Express (Thermo Fisher Scientific) respectively. Cells were resuspended at a concentration of 500.000 cells/mL of PBS/0.4% BSA/0.5 M EDTA. The percentage of Venus positive cells was analyzed with a FACSCanto flow cytometer (Becton Dickinson) equipped with a DIVA software. 10 μg/mL 7-aminoactinomycin D (Sigma-Aldrich) was used for dead cells exclusion. The same procedure was applied for primary CRC spheroids transduced with the TOP-GFP.mCherry construct treated with NanoFEN, oxaliplatin and 5-fluorouracil for in vitro and ex vivo experiments.

### Measurement of intracellular ROS levels

The intracellular generation of ROS was measured using the oxidation-sensitive fluorescent dye 5-chloromethylfluorescein diacetate (CM-H_2_DCFDA), with the conversion of CM-H_2_DCFDA to dichlorofluorescein (DCF) assessed as previously described^54^. An equal number of dissociated spheroid cells (2.5 × 10^5^) were seeded in 6-well cell culture plates. Briefly, each cell line was incubated with NanoFEN at IC50 concentration and pre-treated for 1 h with the antioxidant N-Acetyl-Cysteine (NAC). At different times, the cells were washed twice with pre-warmed Phosphate Saline Buffer (PBS) and then incubated in the same buffer containing 5 μM CM-H_2_DCFDA with DMSO alone at 37 °C. The fluorescence intensity of DCF was measured at 527 nm emission wavelength after excitation at 492 nm at 1 h intervals for up to 48 h using a Flow Cytometer Accuri (Becton Dickinson). An increase in fluorescence intensity as arbitrary units indicated the net generation of intracellular ROS.

### Western blotting

Immunoblotting was performed as previously described^55^. Briefly, whole cell extracts were obtained by incubating the cells in lysis buffer supplemented with protease and phosphatase inhibitor cocktail. Lysates concentration was determined and equal amounts of proteins were loaded on a 4–12% precast gel (Thermo Fisher Scientific) and transferred to nitrocellulose membranes. Blots were blocked with TBST 5% non-fat dry milk (Bio-Rad Laboratories, Hercules, CA, USA) and incubated overnight at 4 °C with primary antibodies (described in the Antibodies and Reagents section) then incubated for 45 min with secondary HRP-conjugated antibodies dissolved in TBST/1% BSA. Chemiluminescent signals were detected with Amersham ECL prime or select western blotting detection reagent (GE Healthcare Life Sciences, Barrington, IL, USA). Images were taken and analyzed with Bio-Rad ChemiDoc Imagers (Bio-Rad Laboratories).

### Viability assay

Cell viability upon treatment with NanoFEN, zVAD, NAC, 3-MA, Myriocin, PPMP, GT11, SA and Rapamycin was determined by CellTiter-Glo luminescent cell viability assay (Promega, Madison, WI, USA) according to the manufacturer’s directions. Briefly, lung and colorectal spheroids were dissociated mechanically or with TrypLE Express (Thermo Fisher Scientific) respectively and seeded 3 × 10^3^ per well in 96-well plates (three replicates per experimental point) in serum-free medium, and incubated in a humidified atmosphere at 37 °C, 5% CO_2_. Cells were treated with the respective compounds and analysed after the appropriate time period as described in detail in Figure Legends. Luminescence was detected with a DTX880 multimode microplate reader (Beckman Coulter, Brea, CA, USA).

### Statistical analysis

Analyses were performed using GraphPad Prism version 4.0 for Windows (GraphPad Software) with non-paired double-tailed t-test (after verifying normal distribution of the population with Shapiro–Wilk test) or with one-way ANOVA where appropriate. Results are presented as the mean ± standard deviation (SD) or standard error of the mean (SEM) where appropriate. Statistical significance is expressed as *P < 0.05; **P < 0.01 and ***P < 0.001.

## Supplementary information

Supplemental Figures

Supplementary Table 1
