# Supplementary material for: A new bioavailable fenretinide formulation with antiproliferative, antimetabolic, and cytotoxic effects on solid tumors
Source: Cell Death Dis. 2019 Jul 23;10(7):529. doi: 10.1038/s41419-019-1775-y (PMC6646369; doi:10.1038/s41419-019-1775-y)
Supplement: Supplementary file 1 — Supplemental Figures [file 41419_2019_1775_MOESM1_ESM.pdf]

A

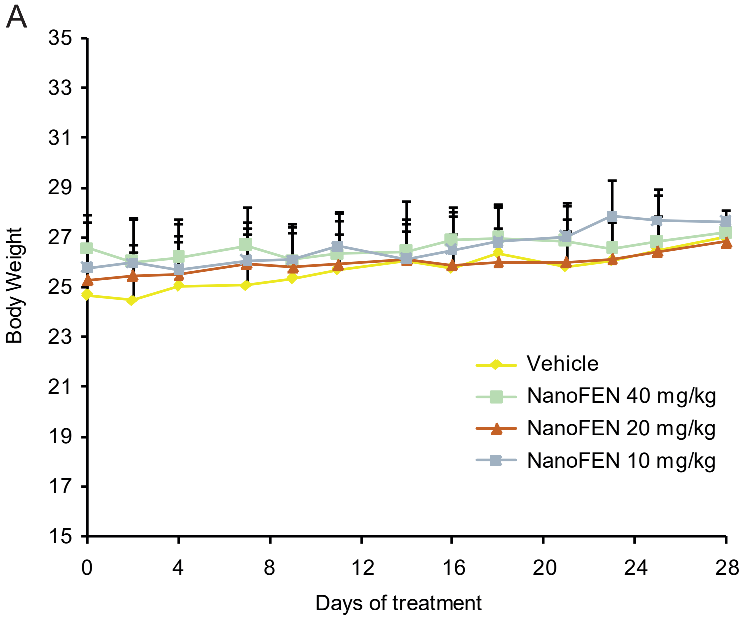

B

	ALT (U/L)	AST (U/L)	Bil (mg/dl)
**CTRL**	84,0 ± 17,35	24,3 ± 4,16	0,4 ± 0,05
**NanoFEN 40 mg/kg**	122,3 ± 32,35	35,0 ± 2,65	0,5 ± 0,13
**NanoFEN 20 mg/kg**	99,0 ± 12,77	28,7 ± 4,73	0,4 ± 0,19
**NanoFEN 10 mg/kg**	109,3 ± 6,81	35,3 ± 4,93	0,3 ± 0,11

C

**Parameters**		**Groups**	
		**Vehicle**	**NanoFEN**
**RBC**	($x10^6$mcl)	9,4 ± 0,2	9,4 ± 0,1
**HB**	(g/dL)	14,7 ± 0,1	15,2 ± 0,3
**HCT**	(%)	44,7 ± 1,4	44,2 ± 0,6
**MCV**	(fL)	47,5 ± 0,4	46,8 ± 0,2
**MCH**	(pg)	15,6 ± 0,4	15,9 ± 0,2
**MCHC**	(g/dL)	32,9 ± 1,3	34,0 ± 0,3
**CHCM**	(g/dL)	32,5 ± 1,2	32,8 ± 0,98
**CH**	(pg)	15,1 ± 0,05	15 ± 0,05
**HDW**	(g/dL)	2,0 ± 0,04	2,0 ± 0,08
**RDW**	(%)	13,1 ± 0,15	12,8 ± 0,1
**PLT**	($x10^3$mcl)	953,6 ± 97,5	985,3 ± 27,3
**MPV**	(fL)	9,5 ± 0,15	8,9 ± 0,8
**PCT**	(%)	0,9 ± 0,13	0,9 ± 0,03
**PDW**	(%)	15,6 ± 1,18	14 ± 1,9
**Leukocytes**	(%)	4,5 ± 0,2	5,7 ± 1,4
**Lymphocytes**	(%)	71,3 ± 1,5	71,6 ± 3
**Monocytes**	(%)	3 ± 2,6	2 ± 1

Supplementary Figure 1. Study of toxic effects of NanoFEN *in vivo* on body weight and hematological parameters. (A) Graph represents body weight variation of mice treated with intravenous NanoFEN at the indicated doses. (B, C) Evaluation of liver subacute toxicity and hematological values of mice treated with NanoFEN at the indicated doses using biochemical parameters of ALT, AST, Bilirubin and blood cell count.

Supplementary Figure 1

A

Lung cancer cells (ATCC)		
**Name**	**10% serum IC50**	**1% serum IC50**
H292 (MEC)	29	24
Calu1 (EC)	35,6	30,5
H460 (LCLC)	27,41	22,14
H1299 (NSCLC)	19	14
H1650 (BAC)	32,12	29
Calu3 (AC)	30,22	25,43
A549 (AC)	30,62	22,2
H1975 (AC)	15,6	9,3
H1781 (AC)	33,7	27,2

Lung cancer spheroid cells	
**Name**	**IC50**
LCSC1	4,3
LCSC2	3,8
LCSC3	0,1
LCSC4	0,9
LCSC5	4,2
LCSC6	4,9

B

Colon cancer cells (ATCC)		
**Name**	**10% serum IC50**	**1% serum IC50**
SW620 (AC)	21,45	5,6
SW480 (AC)	21,81	4,9
HT29 (AC)	10,85	3,5

Colorectal spheroid cells	
**Name**	**IC50**
CSC 1	2,4
CSC 6	0,5
CSC 7	0,2
CSC 8	0,5
CSC 9	1,9
CSC 10	0,2
CSC 11	0,4
CSC 13	2,1
CSC 14	5,4
CSC 15	1
CSC 16	0,8
CSC 17	3,2
CSC 18	1
CSC 19	20
CSC 20	3,7
CSC 21	2,4
CSC 22	1,5
CSC 23	1,4
CSC 25	1,6
CSC 26	5,5
CSC 28	2,3

C

Melanoma cancer cells (ATCC)	
**Name**	**IC50**
A-375	16,9
MeWo	24,5
SK-MEL-28	19,2

Primary melanoma cells	
**Name**	**IC50**
Mel 1	2,6
Mel 3	1,3
Mel 8	1,9

D

Breast cancer cells (ATCC)	
**Name**	**IC50**
MCF7 (AC)	10,2
SK-BR (AC)	8,2
MDA-MB-231 (AC)	14,1

Breast cancer spheroid cells	
**Name**	**IC50**
BCSC105	0,2
BCSC208	0,3
BCSC608	0,4
BCSC709	0,3

E

GBM (ATCC and Sigma- Aldrich)	
**Name**	**IC50**
U251	5,8
U87-MG	5,9
T98MG	5,4

Primary GBM cells	
**Name**	**IC50**
I	0,5
M	3,3
N	0,5
Q	4,9

F

Sarcoma spheroid cells	
**Name**	**IC50**
4540 (Ewing sarcoma)	0,2
LS1P (liposarcoma)	0,9
4052 (chondrosarcoma)	0,9
3844B (osteosarcoma)	0,8

G

Hematological tumor cells (ATCC)	
**Name**	**IC50**
K562 (CML)	17,4
CEM/C1 (LLA)	7,2
Jurkat (acute T cell leukemia)	3,9
TF-1 (erythroleukemia)	1,5

Supplementary Figure 2. Effect of NanoFEN on ATCC cell lines and primary spheroid lines from multiple solid tumors. (A) IC50 of NanoFEN determined in lung cancer ATCC lines in the presence of 10% serum or 1% serum, or in primary lung cancer spheroid cells (LCSC). (B) IC50 of NanoFEN determined in CRC ATCC lines in the presence of 10% serum or 1% serum, or in primary CRC spheroid cells (CSC) (C) IC50 of NanoFEN determined in melanoma ATCC lines in the presence of 10% serum, or in primary melanoma cells (Mel). (D) IC50 of NanoFEN determined in breast cancer ATCC lines in the presence of 10% serum, or in primary breast cancer spheroid cells (BCSC). (E) IC50 of NanoFEN determined in glioblastoma (GBM) ATCC and Sigma-Aldrich cell lines in the presence of 10% serum, or in primary GBM cells. (F) IC50 of NanoFEN determined in primary sarcoma spheroid cells. (G) IC50 of NanoFEN determined in leukemic ATCC lines in the presence of 10% serum.

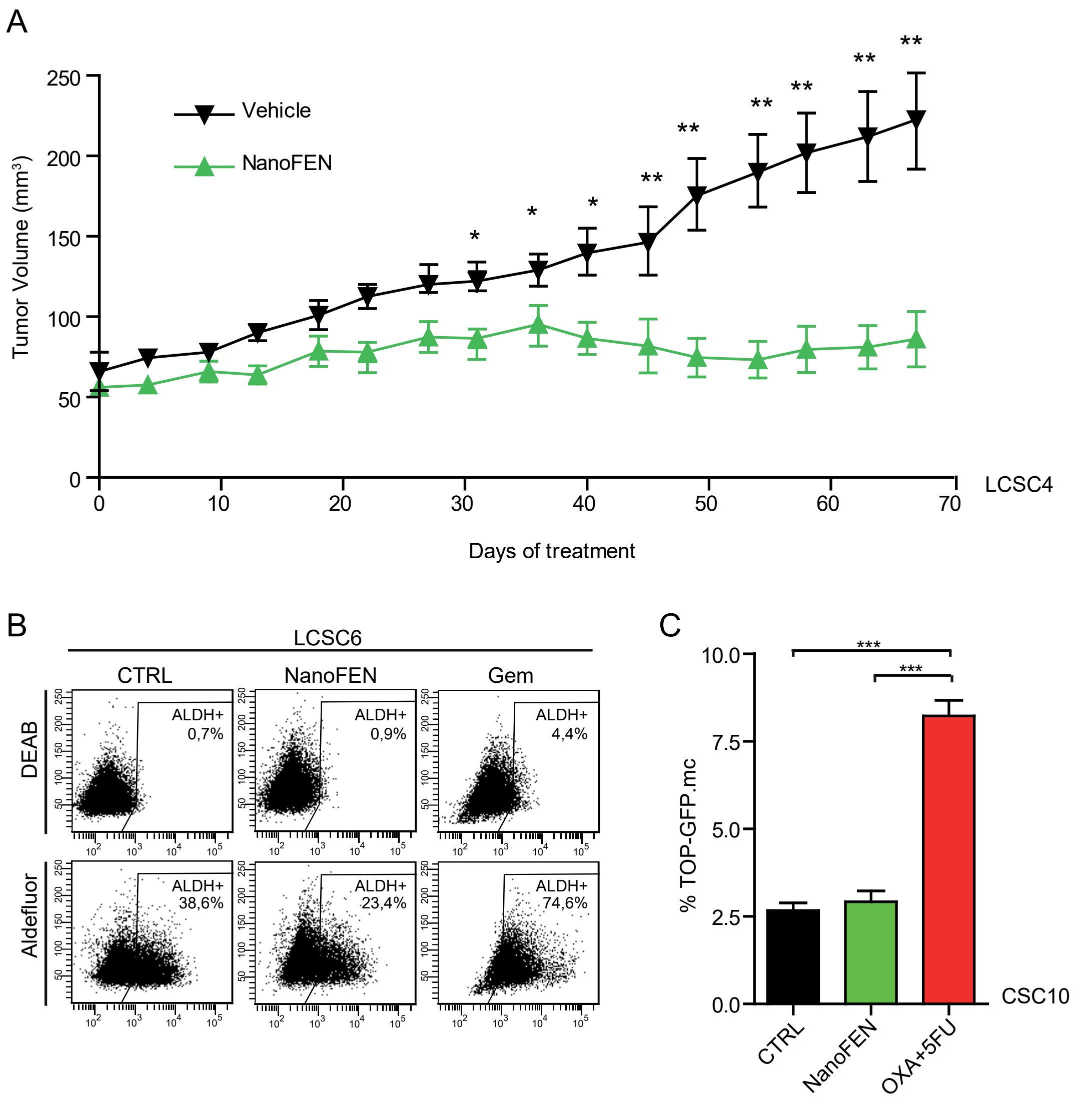

Supplementary Figure 3. Long-term antitumor effect of NanoFEN and effects on cancer stem cell content. (A) Volume of xenografts derived from LCSC4 treated with vehicle (black downward triangles) and treated with NanoFEN 30mg/Kg/week (green upward triangles). Values represent mean ± SEM of three independent experiments. * P<0.05, ** P<0.01 from one-way ANOVA and Bonferroni post-test. (B) Representative cytofluorimetric analysis of Aldefluor activity on LCSC6 treated with NanoFEN at IC50 dose (Supplementary Figure S1 A) and Gemcytabine 25 μM (C) Percentage of TOP-GFP.mc cells present in xenografts derived from CSC10 after 2 weeks of treatment with vehicle only (CTRL), NanoFEN or chemotherapy (OXA+5FU). Data shown are the mean ± SD of three independent experiments. *** P<0.001, from two-tailed t-test.

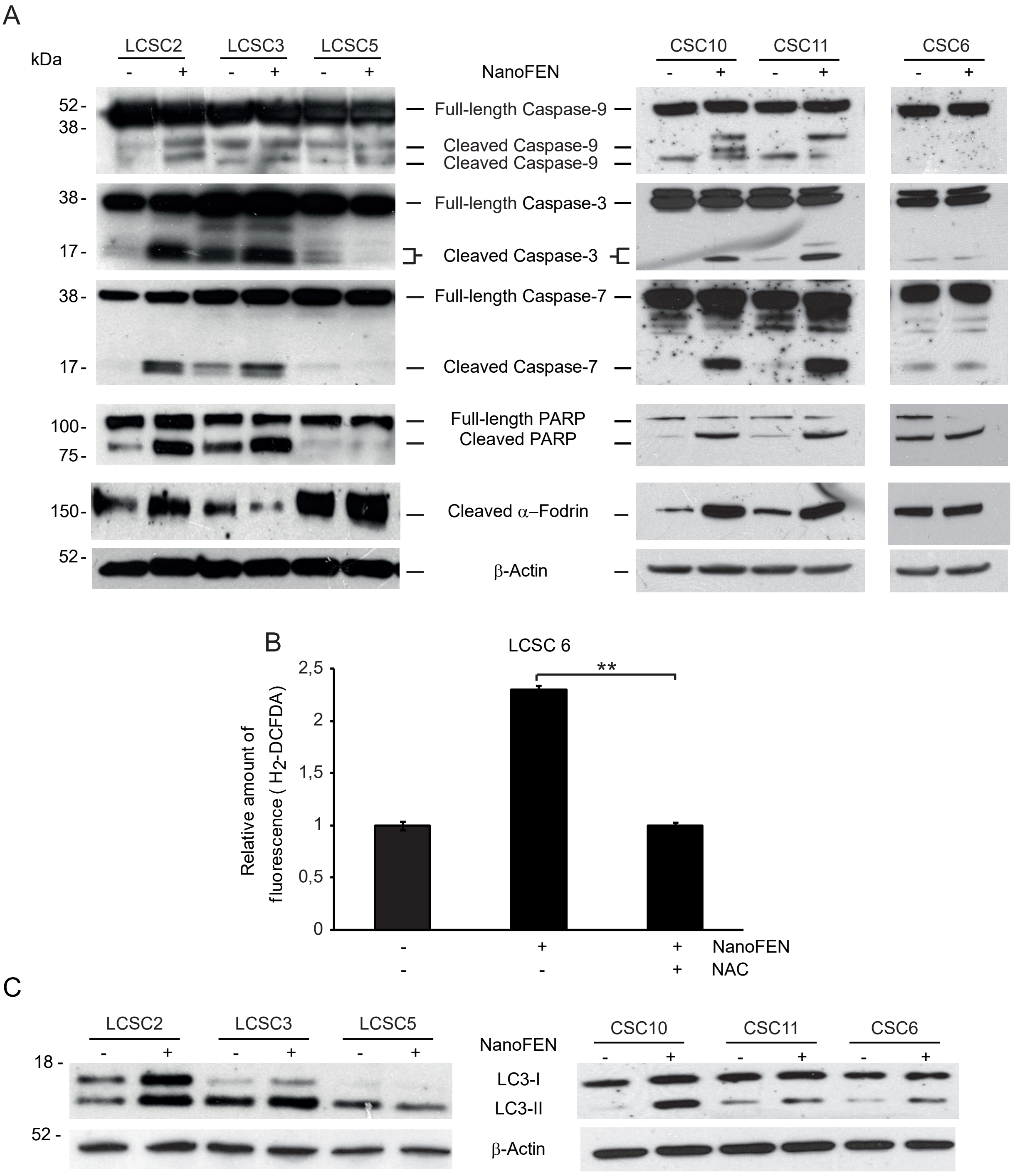

Supplementary Figure 4. Cell death pathways activated by NanoFEN. (A) Immunoblot analysis of cell death-related proteins Caspase-9, -3 and -7, PARP and α-fodrin on LCSC2, LCSC3 and LCSC5 (left panel) and CSC10, CSC11, CSC6 (right panel) untreated and treated with NanoFEN at IC50 dose (Supplementary Fig.2A, B) for 48 hours. (B) Production of reactive oxygen species (ROS) was measured by flow cytometer by using 5μM of H_2CMFDA in LCSC6 pretreated with N-acetyl-cysteine (NAC) 2mM for 1h and after with NanoFEN 4,9μM for 48 hours. Values represent mean ± SD of three independent experiments. ** P<0.01from two-tailed t-test. (C) Immunoblot analysis of autophagy-related marker LC3 I-II on LCSC2, LCSC3, LCSC5 (left panel) and CSC10, CSC11, CSC6 (right panel) untreated and treated with NanoFEN at IC50 dose (Supplementary Fig. 2A, B) for 48 hours.

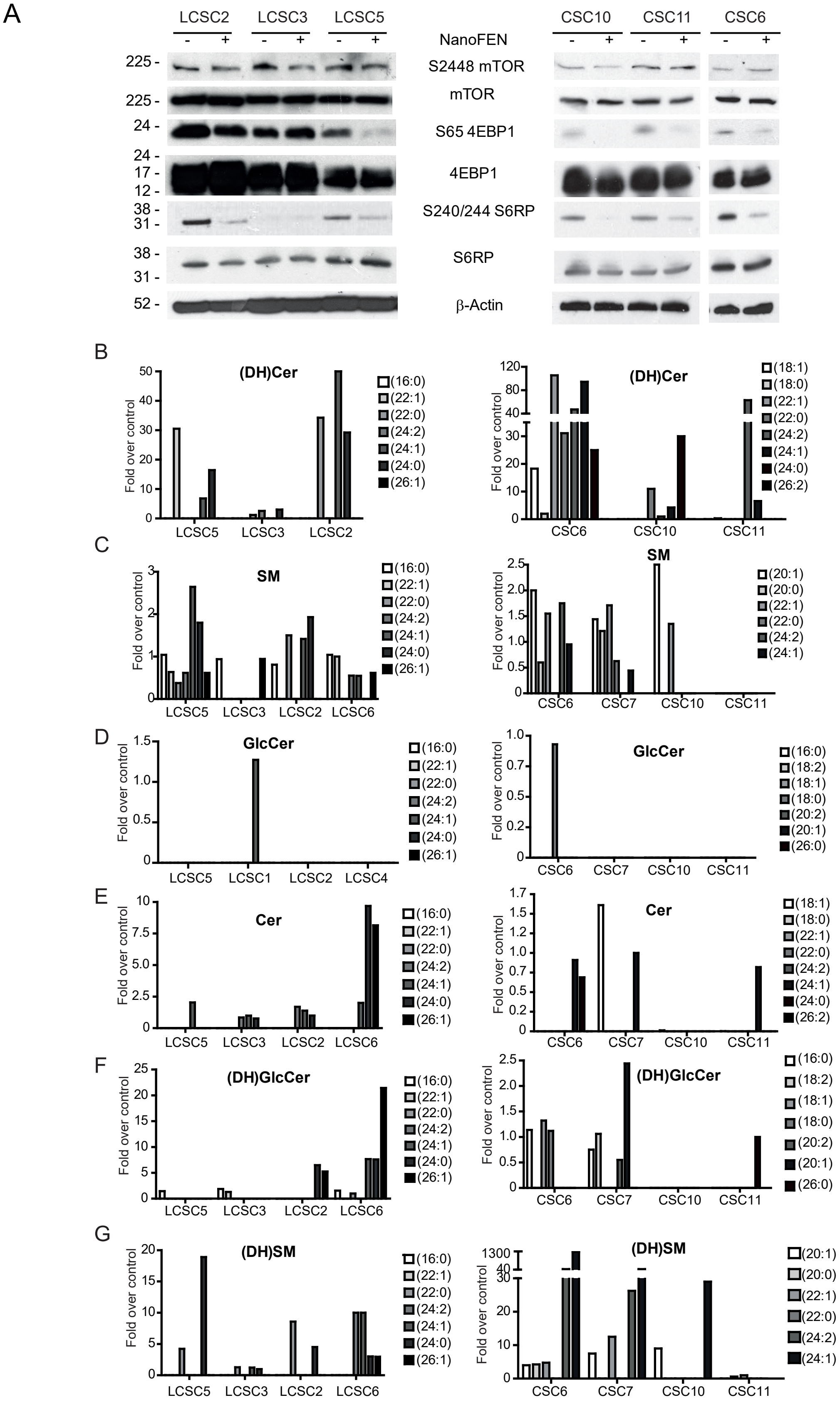

Supplementary Figure 5. NanoFEN alters lipid metabolism. (A) Immunoblot analysis of proteins involved in metabolic process on LCSC2, LCSC3, LCSC5 (left panel) and CSC10, CSC11, CSC6 (right panel) untreated and treated with NanoFEN at IC50 dose (Supplementary Fig. 2A, B) for 48 hours. (B-G) Graphs represent individual dihydroceramides composition of LCSC5, LCSC3, LCSC2 (left side) and CSC6, CSC7, CSC10, CSC11 (right side) treated with NanoFEN at their IC50 dose (Supplementary Fig. 2A, B) for 24 hours were identified by acyl chain, normalized to control and plotted as fold change.

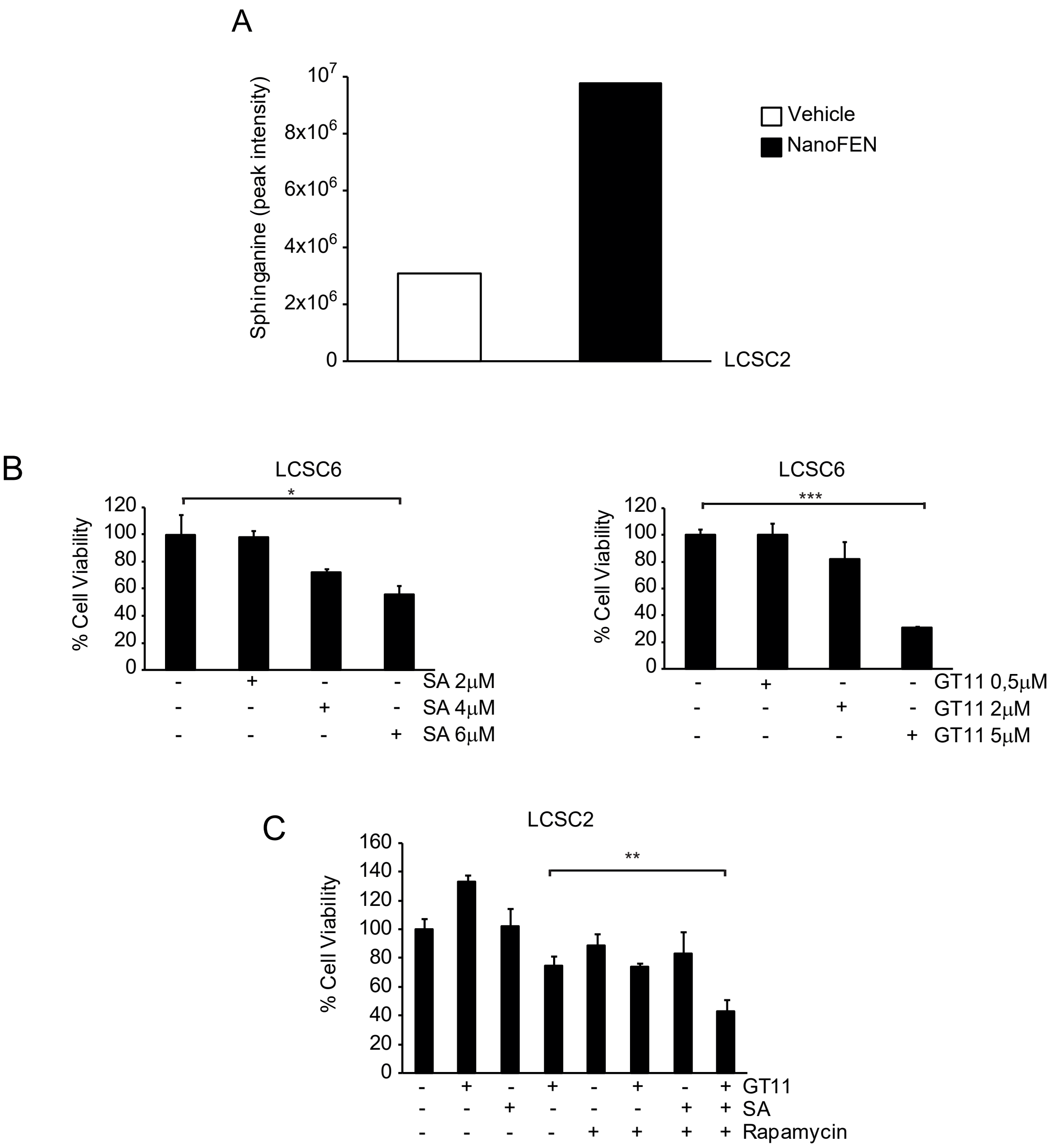

Supplementary Figure 6. Evaluation of lipid metabolism and cell viability *in vivo* and *in vitro*. (A) Sphinganine peak intensity in LCSC2 derived xenografts treated for 3 weeks with NanoFEN (30mg/kg/week) or vehicle. (B) Cell viability of LCSC6 treated with sphinganine (SA) (left panel) and dihydroceramide desaturase inhibitor (GT11) (right panel) at the indicated doses for 48 hours. Values represent mean ± SD of three independent experiments.* $P<0.05$, *** $P<0.001$, from two-tailed t-test. (C) Cell viability of LCSC2 (left panel) treated with dihydroceramide desaturase inhibitor (GT11) 0,5μM plus sphinganine (SA) 2μM plus mTOR pathway inhibitor Rapamycin 10nM for 48 hours. Values represent mean ± SD of three independent experiments. ** $P<0.01$from two-tailed t-test.
